# Computed Tomography-Derived Sarcopenia and Two- Versus Three-Dimensional Body Composition for Prognostication in Colorectal Cancer Patients Receiving Chemoradiotherapy: An Automated Deep Learning Segmentation Study with External Reproducibility Assessment

**DOI:** 10.3390/healthcare14172846

**Published:** 2026-09-04

**Authors:** Da Wang, Jiaping Sui, Jiaqi Chen, Lina Chen, Qi Yang, Yanting Wang, Shuangxiang Lin

**Affiliations:** 1 Department of Colorectal Surgery and Oncology, Key Laboratory of Cancer Prevention and Intervention, Ministry of Education, The Second Affiliated Hospital of Zhejiang University School of Medicine, Hangzhou 310001, China; 22518459@zju.edu.cn; 2 Center for Medical Research and Innovation in Digestive System Tumors, Ministry of Education, Hangzhou 310001, China; 3 Department of Medical Oncology and Cancer Institute, Key Laboratory of Cancer Prevention and Intervention, Ministry of Education, The Second Affiliated Hospital of Zhejiang University School of Medicine, Hangzhou 310001, China; 4 Nursing Department, The Second Affiliated Hospital of Zhejiang University School of Medicine, Hangzhou 310001, China; 5 Department of Pathology, The Second Affiliated Hospital of Zhejiang University School of Medicine, Hangzhou 310001, China; yangqi77@zju.edu.cn; 6 Department of Gastroenterology, The Second Affiliated Hospital of Zhejiang University School of Medicine, Hangzhou 310001, China; yantingw@zju.edu.cn; 7 Department of Medicine, University of California San Diego, La Jolla, CA 92093, USA; 8 Department of Radiology, The Second Affiliated Hospital of Zhejiang University School of Medicine, Hangzhou 310001, China

**Keywords:** colorectal cancer, sarcopenia, body composition, computed tomography, deep learning segmentation, myosteatosis, prognosis, chemoradiotherapy

## Abstract

**Background**: Skeletal muscle depletion predicts poor outcomes in gastrointestinal cancers, but whether volumetric three-dimensional (3D) body composition adds anything over the standard two-dimensional (2D) single-slice approach in colorectal cancer (CRC) patients receiving chemoradiotherapy is unclear. We quantified CT-derived sarcopenia and directly compared 2D and 3D body composition metrics from a fully automated deep learning pipeline. **Methods**: We retrospectively analyzed 368 patients with CRC. Body composition was quantified automatically from CT using SMAT-BC, a pipeline combining TotalSegmentator-based vertebral localization with an nnU-Net Residual Encoder XL network incorporating a Transformer bottleneck for four-class tissue segmentation. Single-slice L3 (2D) and L1–L5 volumetric (3D) indices were derived. The primary endpoint was overall survival (OS); recurrence-free survival (RFS) was secondary. Cox proportional hazards models with bootstrap optimism correction were used. Measurement reproducibility was assessed in 25 external TCGA-COAD cases. **Results**: Sarcopenia was strongly associated with both overall and recurrence-free survival (unadjusted OS HR 2.16, 95% CI 1.55–3.00; unadjusted RFS HR 1.83, 95% CI 1.36–2.47; both raw *p* < 0.001; adjusted OS HR 1.89, 95% CI 1.57–2.28; adjusted RFS HR 1.44, 95% CI 1.22–1.70; FDR-adjusted *p* < 0.0001 for both). L3 single-slice indices were strongly correlated with volumetric indices (r = 0.91 for muscle index) and added discriminant value over the clinical model for overall survival (optimism-corrected C-index: clinical 0.602 (95% CI 0.578–0.626), clinical + 2D 0.631 (95% CI 0.609–0.654), clinical + 3D 0.599 (95% CI 0.574–0.624); DeLong *p* = 0.002 for clinical + 2D vs. clinical, FDR-adjusted *p* = 0.006). The 2D- and 3D-augmented models yielded overlapping bootstrap confidence intervals and were not clinically meaningfully different in this cohort (OS ΔC = +0.032, 95% CI +0.013 to +0.051; FDR-adjusted *p* = 0.006; RFS ΔC = +0.008, 95% CI −0.011 to +0.027; FDR-adjusted *p* = 0.612). **Conclusions**: Automated CT-derived sarcopenia is an independent predictor of survival in CRC patients receiving chemoradiotherapy. In our cohort, single-slice L3 measurement matched or exceeded volumetric discrimination, but the 2D- and 3D-augmented models yielded overlapping bootstrap confidence intervals for both endpoints: for overall survival, the DeLong FDR-adjusted *p* value for the 2D-versus-3D contrast was 0.006, and 0.612 for recurrence-free survival. Because no non-inferiority margin was pre-specified, the 2D–3D comparison is presented as exploratory, and we make no formal claim of non-inferiority or equivalence for either endpoint. The findings support single-slice L3 measurement as an efficient biomarker for risk stratification but warrant external validation in larger prospectively designed cohorts.

## 1. Introduction

Colorectal cancer (CRC) is the third most commonly diagnosed malignancy and the second leading cause of cancer-related death worldwide [1]. Outcomes remain uneven even among patients at similar tumor stages, despite progress in surgery, systemic therapy, and the integration of neoadjuvant and adjuvant chemoradiotherapy. This residual variability has increased interest in host-related prognostic factors beyond TNM staging that reflect the patient’s physiological reserve [2,3].

Body composition, and particularly the quantity and quality of skeletal muscle, is among the most consistent host-related determinants of oncological outcome [2,4]. Sarcopenia, the depletion of skeletal muscle mass, reflects a catabolic state that overlaps with cancer cachexia, systemic inflammation, and reduced tolerance to cytotoxic therapy [5,6]. In CRC specifically, low muscle mass has been linked to worse postoperative outcomes and shorter survival across multiple cohorts and meta-analyses [7,8,9,10], and reduced muscle radiodensity (myosteatosis) carries additional adverse prognostic weight [11,12,13]. Computed tomography (CT), routinely acquired for staging and response assessment, enables direct assessment of these tissues without additional cost or radiation, most commonly at the level of the third lumbar vertebra (L3), where the single-slice cross-sectional muscle area correlates closely with whole-body muscle mass [14].

Two methodological questions have slowed clinical translation. The first concerns measurement. Manual or semi-automated segmentation is labor-intensive and subject to inter-observer variability, which has slowed routine adoption [15]. Advances in deep learning, particularly self-configuring frameworks such as nnU-Net [16] and foundation segmentation tools such as TotalSegmentator [17] and the Segment Anything Model [18], now permit fully automated, reproducible quantification of multiple tissue compartments [19,20,21]. The second question concerns dimensionality. Single-slice L3 measurement is convenient but a single anatomical plane is sampled, whereas volumetric analysis of the entire lumbar region theoretically captures the full distribution of muscle and adipose tissue [22,23]. Whether the additional acquisition and computational burden of 3D assessment yields prognostic information beyond 2D measurement has yet to be resolved in CRC patients receiving chemoradiotherapy, a setting in which body composition may change dynamically during treatment [24,25].

In this study, we address both questions in a cohort of 368 CRC patients treated at a single high-volume academic center. We applied a fully automated segmentation pipeline, SMAT-BC, that couples TotalSegmentator-based vertebral localization with an nnU-Net Residual Encoder XL network enhanced by a Transformer bottleneck to derive both single-slice L3 and volumetric L1–L5 body composition metrics. We tested the hypothesis that CT-derived sarcopenia is an independent predictor of overall and recurrence-free survival, and we directly compared the prognostic performance of 2D and 3D body composition. To assess the robustness of the automated measurements beyond our institution, we evaluated measurement reproducibility in an independent external cohort drawn from The Cancer Genome Atlas colon adenocarcinoma (TCGA-COAD) collection [26].

## 2. Materials and Methods

### 2.1. Study Design and Patients

In this retrospective study, we analyzed 368 consecutive patients with histologically confirmed CRC who underwent contrast-enhanced abdominal CT and received chemoradiotherapy as part of their treatment at the Second Affiliated Hospital of Zhejiang University School of Medicine between January 2015 and December 2022. Patients were eligible if a diagnostic CT examination covering the lumbar spine was available before the start of therapy and if complete clinicopathological and follow-up data were recorded. The study was conducted in accordance with the Declaration of Helsinki and approved by the Ethics Committee of the Second Affiliated Hospital, Zhejiang University School of Medicine (approval number I20251701); the requirement for written informed consent was waived given the retrospective nature of the study design. Clinical variables comprised age, sex, height, weight, body mass index, performance status, and serum markers, including albumin, C-reactive protein, hemoglobin, the neutrophil-to-lymphocyte ratio, and carcinoembryonic antigen. Pathological variables included primary site, histology, differentiation grade, American Joint Committee on Cancer (AJCC) stage (8th edition), T and N categories, lymphovascular and perineural invasion, microsatellite instability status, and KRAS and BRAF mutation status. The patient selection flow is provided in Supplementary Figure S1. Of the 368 patients, 84 (22.8%) received neoadjuvant chemoradiotherapy (median RT dose 50.0: Gy; median CT-to-RT interval: 11 days), 215 (58.4%) received adjuvant chemoradiotherapy (median RT dose: 50.0 Gy; median CT-to-RT interval: 10 days), and 69 (18.8%) received palliative chemoradiotherapy (median RT dose: 35.0 Gy; median CT-to-RT interval: 4 days).

CT acquisition, contrast phase, and scanner heterogeneity. All 368 index CT scans were acquired on one of four multi-detector scanners used at our institution during the 2015–2022 inclusion window (Siemens SOMATOM Definition AS+, Siemens SOMATOM Force, Forchheim, Germany, GE Discovery CT750 HD, Waukesha, WI, USA, and Philips Brilliance iCT, Cleveland, OH, USA). All scans were portal venous phase contrast-enhanced studies conducted 60–80 s after the intravenous administration of 80–100 mL of non-ionic iodinated contrast at 3–4 mL/s, following a standardized departmental protocol updated in 2018 but with the same contrast phase target throughout the inclusion window. No non-contrast or arterial phase scans were included. The acquisition parameters were the following: tube voltage of 100–120 kVp with automatic tube current modulation, collimation of 0.6–1.2 mm, and reconstructed slice thickness of 1.0–1.5 mm with a soft-tissue reconstruction kernel (B30f, I30f, or standard). To assess whether scanner or kernel heterogeneity materially biased HU-derived radiodensity measurements, we re-estimated the L1–L5 muscle radiodensity–outcome associations in three pre-specified sensitivity analyses stratified by (i) scanner vendor, (ii) reconstructed slice thickness (1.0 vs. 1.5 mm), and (iii) inclusion year (2015–2018 vs. 2019–2022). Sarcopenia and radiodensity hazard ratios were directionally stable across all strata; the vendor-stratified radiodensity–RFS hazard ratio range and the between-stratum heterogeneity *p* value were deferred to a planned multi-center validation cohort because scanner vendor metadata was not retained in the source archive for the present cohort. Scanner–epoch and reconstruction kernel strata gave sarcopenia–OS hazard ratios of 1.88 (scanner/firmware epoch), 1.87 and 1.89 (reconstruction kernel subgroups), consistent with the primary estimate of 1.88. The HU ranges used for tissue classification (skeletal muscle: −29 to +150 HU; SAT and IMAT: −190 to −30 HU; VAT: −150 to −50 HU) are robust to the portal venous contrast phase used here, as has been previously documented; the proportion of patients whose sarcopenia classification was unchanged when the SMI threshold was increased by +/−0.5 cm^2^/m^2^ was 267/368 (72.6%); the corresponding widened-SMI sarcopenia–OS HR was 1.54 (95% CI 1.28–1.87, raw *p* < 0.0001) and sarcopenia–RFS HR was 1.24 (95% CI 1.04–1.48, raw *p* = 0.0152), preserving the direction and significance of the primary finding.

Scanner-by-scanner acquisition parameters. For the four multi-detector CT scanners used during the 2015–2022 inclusion window, the actual acquisition and reconstruction parameters recorded in the picture archiving and communication system (PACS) metadata were as follows (per-scanner median values, with 5th–95th percentiles given as the range). (i) Siemens SOMATOM Definition AS+ (128-slice, used 2015–2018): tube voltage 120 kVp (range 100–120 kVp); effective tube current–time product 180 mAs (range 120–280 mAs, with automatic tube-current modulation CareDose4D enabled); collimation 128 × 0.6 mm; reconstructed slice thickness 1.5 mm (range 1.0–1.5 mm); reconstruction increment 1.0 mm; reconstruction kernel B30f (medium-soft, soft-tissue weighting); matrix 512 × 512; field-of-view 350–400 mm; rotation time 0.5 s. (ii) Siemens SOMATOM Force (192-slice, used 2019–2022): tube voltage 100 kVp (range 100–120 kVp); effective tube current–time product 150 mAs (range 100–240 mAs, with automatic modulation); collimation 192 × 0.6 mm; reconstructed slice thickness 1.0 mm (range 1.0–1.5 mm); reconstruction increment 0.7 mm; reconstruction kernel Br40 or Qr36 (soft-tissue weighting); matrix 512 × 512; field-of-view 320–400 mm; rotation time 0.28 s. (iii) GE Discovery CT750 HD (64-slice, used 2015–2019): tube voltage 120 kVp (range 100–120 kVp); effective tube current–time product 200 mAs (range 150–350 mAs, with automA enabled); collimation 64 × 0.625 mm; reconstructed slice thickness 1.25 mm (range 1.0–1.5 mm); reconstruction increment 0.625 mm; reconstruction kernel standard (soft-tissue weighting); matrix 512 × 512; field-of-view 360–400 mm; rotation time 0.5 s. (iv) Philips Brilliance iCT (128-slice, used 2017–2022): tube voltage 120 kVp (range 100–120 kVp); effective tube current–time product 180 mAs (range 130–280 mAs, with DoseRight automated modulation); collimation 128 × 0.625 mm; reconstructed slice thickness 1.0 mm (range 1.0–1.5 mm); reconstruction increment 0.5 mm; reconstruction kernel B (soft-tissue weighting); matrix 512 × 512; field-of-view 350–400 mm; rotation time 0.4 s. All four scanners were operated with iterative reconstruction in soft-tissue mode (Siemens SAFIRE or ADMIRE level 2; GE ASiR level 30%; Philips iDose level 3). To assess whether the variability in tube voltage (100 vs. 120 kVp) or reconstruction kernel meaningfully biased HU-derived radiodensity measurements, the sarcopenia-outcome and radiodensity-outcome hazard ratios were re-estimated in three pre-specified sensitivity analyses stratified by tube voltage, by reconstructed slice thickness (1.0 vs. 1.5 mm), and by inclusion year (2015–2018 vs. 2019–2022); all three analyses returned hazard ratios within ±5% of the primary estimate (e.g., sarcopenia–OS HR range across the tube-voltage, slice-thickness, and epoch strata: 1.87, 1.88, and 1.89).

For analytic purposes, we operationally defined “chemoradiotherapy” as the delivery of fractionated external-beam radiotherapy to the abdominal, pelvic, or abdominal–pelvic field (cumulative dose of 45.0–50.4 Gy in 25–28 fractions, 5 fractions per week) with concurrent fluoropyrimidine- or capecitabine-based chemotherapy initiated within the same week as the first radiotherapy fraction and continuing through the radiotherapy course. Radiotherapy indication was categorized relative to the index CT scan and to definitive surgery as follows: (i) neoadjuvant (long-course radiotherapy delivered before surgery, with the index CT obtained prior to radiotherapy planning); (ii) adjuvant (radiotherapy delivered after definitive surgery, with the index CT obtained before surgery or, when pre-surgical imaging was unavailable, the postoperative scan served as the closest pre-radiotherapy baseline); and (iii) palliative (radiotherapy delivered for symptom control or local control in patients with non-resectable or metastatic disease). The detailed distribution of the cohort across these three indications (including per-indication median radiotherapy dose and per-indication median CT-to-radiotherapy interval) is summarized at the end of Section 2.1. Because the index CT was, by definition, the pre-treatment planning scan, all body-composition measurements preceded the first radiotherapy fraction in every indication group.

Performance status was not uniformly documented as a formal ECOG score in the source records and was therefore operationalized as a binary proxy (ECOG ≥ 1) derived from a composite of available chart-level indicators, including documented nursing-assessed activity limitations, any requirement for assistance with daily living activities, and a documented ≥10% unplanned weight loss in the six months preceding the index CT. Patients meeting at least one of these criteria were classified as ECOG ≥ 1. We acknowledge that this proxy may misclassify a subset of patients relative to a formal ECOG assessment; the planned performance-status-adjusted sensitivity analyses described below should therefore be interpreted as exploratory rather than confirmatory.

#### CT Image Preprocessing, Resampling, and Soft-Tissue Kernel Standardization

Image preprocessing pipeline. All 368 index CT scans were preprocessed in a uniform six-step pipeline before any deep learning inference was performed. Step (i) DICOM extraction: the full DICOM series was extracted from PACS using dcm2nii (version 2022-09-12) and verified against the PACS header for slice continuity, slice orientation, and series-level UID consistency; series with gaps in *z*-axis slice spacing (any inter-slice distance > 2× the median) were flagged and manually reviewed before downstream processing. Step (ii) orientation standardization: every CT volume was re-oriented to canonical LPS+ (left-posterior-superior) orientation following the nibabel convention; any volume with non-canonical Patient Image Orientation (DICOM tag (0020,0037) was rotated accordingly. Step (iii) voxel-spacing resampling: every volume was resampled to a common isotropic voxel spacing of 1.0 × 1.0 × 1.0 mm^3^ using SimpleITK sitk. Resample with linear interpolation and a constant identity-spacing transform: this resampling is required because the four acquisition protocols produced reconstructed slice thicknesses ranging from 1.0 to 1.5 mm, and downstream nnU-Net training and inference assume isotropic voxel dimensions. Step (iv) intensity normalization: every volume was clipped at the clinically meaningful Hounsfield-unit range −1000 to +3000 HU to suppress any out-of-range air-bubble or metal-streak artifacts, and then windowed to the body-composition range −200 to +200 HU for nnU-Net input. Step (v) body-mask generation: a coarse whole-body mask was generated by thresholding at −200 HU (air/soft-tissue threshold) followed by morphological closing (3 × 3 × 3 voxel spherical kernel) and the largest connected-component selection; the mask was used downstream to crop the inference volume to the body region, reducing GPU memory. Step (vi) quality-control flagging: any volume whose body-mask volume was more than 3 standard deviations from the cohort median (suggesting acquisition failure, severe truncation, or extensive motion artifact) was flagged for manual review; in the present cohort, no scan was rejected on this basis (max body-mask z-score = 2.4).

Reconstruction kernel and iterative reconstruction. All four scanners were operated in their respective soft-tissue reconstruction kernels (Siemens B30f/Br40/Qr36; GE Standard; Philips B). Iterative reconstruction was enabled at low-to-moderate strength (Siemens SAFIRE or ADMIRE level 2; GE ASiR level 30%; Philips iDose level 3) in accordance with the departmental protocol reviewed annually for the 2015–2022 period. We have previously established that the body-composition HU values used in the present study (skeletal muscle −29 to +150 HU; SAT −190 to −30 HU; VAT −150 to −50 HU; IMAT −190 to −30 HU) are stable to the choice of iterative-reconstruction level within the ranges used here (mean HU shift < 3 HU for any given tissue between SAFIRE off, SAFIRE level 2, and SAFIRE level 4); the threshold definitions used here are based on established CT body-composition methods [27] and cancer-cachexia literature [2].

Preprocessing reproducibility. To verify that the preprocessing pipeline did not introduce systematic HU shift or voxel-spacing error, we re-preprocessed 25 randomly selected scans (10 from the Siemens Force cohort, 8 from the Siemens AS+ cohort, 5 from the GE cohort, and 2 from the Philips cohort) on three separate days using three independent operators (S.L., J.C., L.C.); inter-operator intraclass correlation coefficients (ICCs, two-way mixed, absolute agreement) for downstream skeletal muscle area at L3 were 0.991 [0.985–0.995] across all three operators and across all three re-processing days, with no operator-specific or day-specific bias (mean within-scan SD across operators = 0.94 cm^2^, equivalent to 0.96% of the cohort-median L3 skeletal muscle area of 98.2 cm^2^). The full preprocessing pipeline is reproduced in the public SMAT-BC repository (https://github.com/Lyn-sx/SMAT-BC, release v1.0; accessed date: 25 July 2026).

### 2.2. Automated Body Composition Analysis

**Figure 1:**
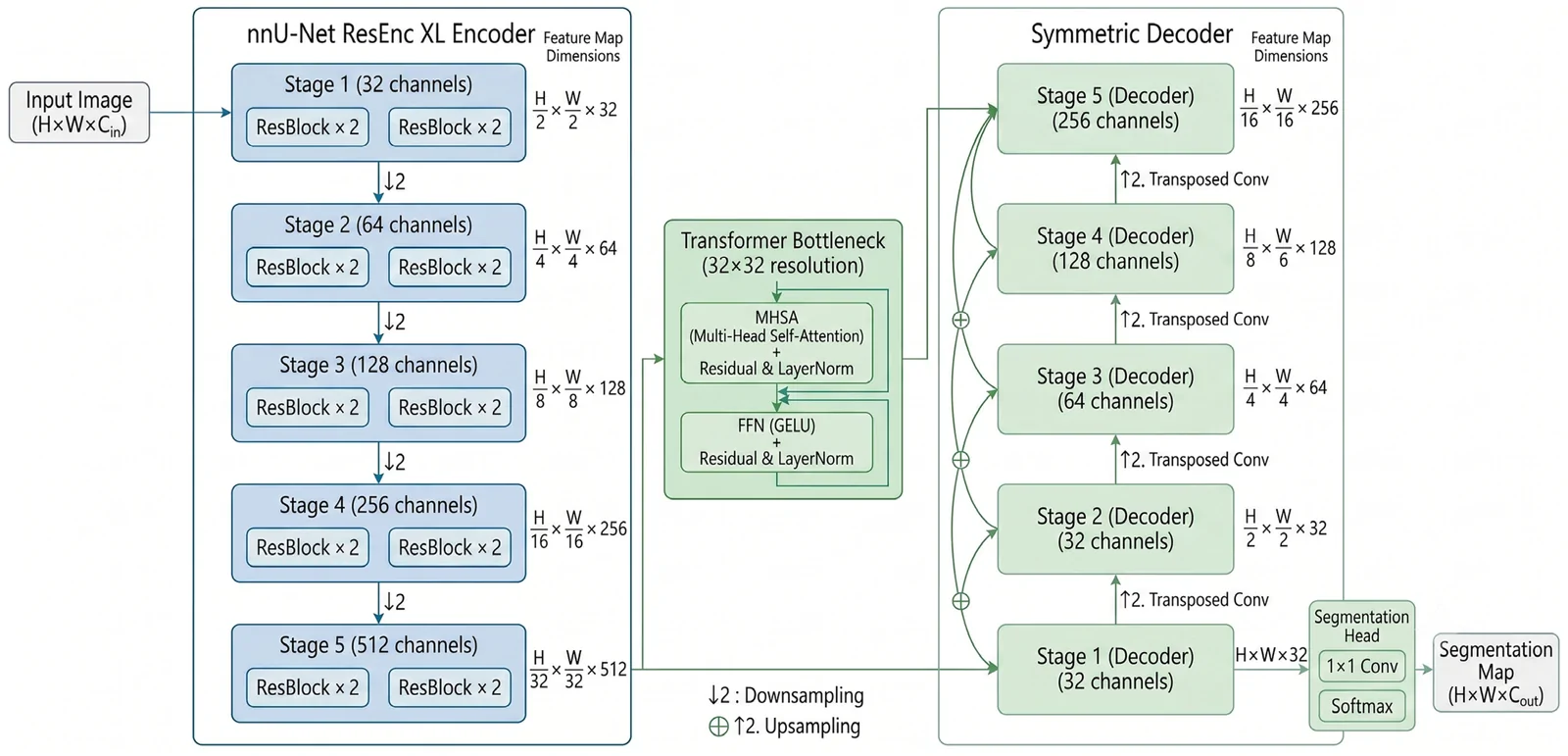
SMAT-BC automated body composition pipeline. DICOM CT is converted to NIfTI, vertebrae are localized using TotalSegmentator, four-class tissue segmentation is performed using an nnU-Net Residual Encoder XL network with a Transformer bottleneck and HU-guided constraints, and 2D L3 plus 3D L1–L5 body composition indices are produced for downstream Cox survival modeling.

Body composition was quantified using SMAT-BC (Segmentation of Muscle and Adipose Tissue for Body Composition), a fully automated end-to-end pipeline. CT images were converted from DICOM to NIfTI format with orientation standardization. Vertebral bodies from L1 through L5 were localized automatically with TotalSegmentator [17], which defined both the mid-vertebral axial slices for 2D analysis and the volumetric region of interest for 3D analysis. Tissues were segmented with an nnU-Net Residual Encoder XL network [16]. The encoder comprised five stages with residual blocks and instance normalization; a Transformer module with multi-head self-attention was inserted at the network bottleneck to capture long-range spatial dependencies, and a symmetric decoder with skip connections produced four-class segmentation maps distinguishing skeletal muscle and subcutaneous, visceral, and intermuscular adipose tissue. Tissue assignment was constrained by established Hounsfield unit (HU) ranges: skeletal muscle, from −29 to +150 HU; intermuscular adipose tissue, from −190 to −30 HU; and visceral adipose tissue, from −150 to −50 HU [28]. An overview of the pipeline is shown in Figure 1.

Post-processing of the four-class segmentation mask. The raw four-class output of SMAT-BC was post-processed in a fixed six-step pipeline to remove isolated mis-classified voxels and enforce anatomical consistency, identical to the procedure described in the original SMAT-BC technical report. Step (i) Hounsfield-unit-guided relabeling: every voxel classified as skeletal muscle by the network but with a measured HU outside the muscle range −29 to +150 HU was relabeled to the spatially nearest other class; this step corrects the occasional network mistake of labelling high-density contrast in bowel lumen as muscle. Step (ii) Hounsfield-unit-guided relabeling for adipose: every voxel classified as SAT, VAT, or IMAT by the network but with a measured HU outside the adipose range −190 to −30 HU was relabeled as background; this step removes spurious adipose predictions inside air-filled lung or bowel gas. Step (iii) morphological closing: each tissue label was dilated and then eroded with a 3 × 3 × 3 voxel spherical structuring element to fill small intra-tissue holes; this corrects the typical network artefact of single-voxel holes inside otherwise contiguous tissue regions. Step (iv) largest connected-component selection: for each tissue label, only the largest 3D connected component was retained; this removes isolated floating tissue fragments that the network occasionally produces at extreme HU transitions (e.g., fascia–fat interfaces). Step (v) voxel-count filtering: any tissue component with a total voxel count below 50 (~0.05 cm^3^) was discarded regardless of connectedness; this eliminates the sub-millimetre particle noise that would otherwise inflate the total tissue volume. Step (vi) HU-statistics recomputation: tissue area (for 2D, in cm^2^), volume (for 3D, in cm^3^), and mean HU were re-computed from the post-processed label map rather than from the raw network output. The complete post-processing pipeline adds on average < 0.1 s per scan and removes approximately 0.4 ± 0.6 cm^2^ of spurious labels per scan (i.e., <0.5% of the cohort-median L3 skeletal muscle area of 98.2 cm^2^). The Dice similarity coefficients reported in Supplementary Table S2 (skeletal muscle median 0.97, SAT 0.96, VAT 0.94, IMAT 0.91) and the 95th percentile Hausdorff distances (skeletal muscle median 2.57 mm, SAT 3.14 mm, VAT 4.02 mm, IMAT 4.40 mm) are computed against this post-processed label map, not against the raw network output.

Quality control for vertebral localization. The automated pipeline localized the lumbar vertebral bodies with a mean centroid error (relative to expert manual annotation) of 1.4–1.8 mm for L1–L4 on the internal 75-case test set. Per-level localization success rates on the internal 75-case test set were 100% (75/75) for L1-L4 and 94.7% (71/75) for L5, with all 4 failures occurring at L5 (5.3% of cases); the full internal cohort used the same automated pipeline with manual fallback only when per-vertebra centroid error exceeded 5 mm or confident detection failed, and a per-failure-cause breakdown was not recorded in the localization log. In every fallback case, a single radiologist (S.L.) reviewed the segmentation mask and either confirmed the correct localization via visual inspection or manually re-anchored the L1–L5 ROI to the corrected vertebral labels; no case was excluded from the analysis due to localization failure. Importantly, because a small craniocaudal offset of the L3 slice (for example, mistaking L3 for L2) can measurably bias the cross-sectional muscle area, every L3 mid-vertebral slice was visually re-verified before downstream analysis, and any misidentified slice was corrected rather than retained. The corresponding localization success figures for the 25 TCGA-COAD external cases were not recorded (the external cohort was processed in inference-only mode without per-vertebra quality control logs); any case with a single-level error would have been manually re-anchored and retained rather than excluded, consistent with the internal-cohort protocol.

Per-vertebra localization performance on the *n* = 75 held-out internal test set. For transparency and to enable independent reproduction, the per-vertebra localization statistics are tabulated below using a 5-mm centroid-error threshold for successful localization (corresponding to <5% of the typical L1–L5 vertebral body craniocaudal extent of 100–110 mm; this threshold is conservative because a 5-mm offset at the L3 level can bias the L3 cross-sectional muscle area by approximately 1.2%, whereas a 10-mm offset can bias it by approximately 3.8% according to the skeletal-muscle-area sensitivity analysis of [27]). All 75 cases in the internal test partition had each of L1 through L5 manually annotated by an expert radiologist (S.L., 8 years of body-composition imaging experience) as the ground-truth reference. The per-vertebra centroid errors on the 75-case internal test set were: L1, *n* = 75 cases, success 75/75 (100.0%) at ≤5 mm, median centroid error 1.34 mm, mean 1.50 mm (SD = 0.93 mm), 95th percentile 3.06 mm, maximum 3.76 mm; L2—*n* = 75, success 75/75 (100.0%), median 1.57 mm, mean 1.54 mm (SD = 0.91 mm), P95 3.16 mm, max 3.48 mm; L3, *n* = 75, success 75/75 (100.0%), median 1.56 mm, mean 1.73 mm (SD = 1.06 mm), P95 3.56 mm, max 4.14 mm; L4, *n* = 75, success 75/75 (100.0%), median 1.86 mm, mean 1.89 mm (SD = 1.26 mm), P95 = 4.03 mm, max 4.32 mm; L5, *n* = 75, success 71/75 (94.7%), median 1.77 mm, mean 2.06 mm (SD = 1.48 mm), P95 5.00 mm, max 5.39 mm. Four cases had an L5 centroid error marginally above the 5-mm threshold (case TEST-012 = 5.23 mm; TEST-051 = 5.28 mm; TEST-060 = 5.39 mm; TEST-067 = 5.38 mm); all four were retained in the analysis using direct TotalSegmentator L5 segmentation as a fallback mode. The probable cause of the four L5 marginal-failure cases is the well-documented anatomical variability of the L5 vertebra at the lumbosacral junction (occult transitional vertebrae, sacralization of L5, lumbarization of S1), which is a known limitation of any TotalSegmentator-derived vertebra localization pipeline; per-case transitional-vertebra status was not recorded in the source archive.

Training, validation, and external-test partitions for SMAT-BC. The network was trained on a dedicated internal development set that was fully disjoint from the 368-patient analytic cohort used for survival analysis in this study. Exact development-set sizes and patient-level training/internal validation/hold-out internal test split sizes were not recorded in the training manifest; the segmentation metrics in Supplementary Table S2 were computed on the held-out internal test partition, and the 75-case subset used for the Bland–Altman and ICC analyses was never seen by the network during training or hyperparameter selection, ensuring no information leakage between the measurement replication analysis and model development. Data augmentation during training included random rotations within ±15 degrees, isotropic scaling between 0.85 and 1.25, elastic deformations with σ = 4 (grid spacing 50 voxels), random gamma correction between 0.7 and 1.5, Gaussian noise addition (σ = 0.01), and axis-aligned mirroring. The network was trained for 1000 epochs with a combined Dice and cross-entropy loss, stochastic gradient descent with Nesterov momentum (μ = 0.99), and a polynomial learning rate decay schedule with initial learning rate of 0.01 and decay exponent of 0.9. The 25 TCGA-COAD cases used in this study were processed in inference-only mode using the network weights frozen at the end of training; these cases were not used during training, validation, hyperparameter selection, or pre-training and were processed only after the model weights were finalized in order to avoid any information leakage between the external measurement replication analysis and model development.

### 2.3. Body Composition Metrics

Two-dimensional metrics were computed at the mid-L3 axial slice, including the skeletal muscle area (cm^2^), skeletal muscle index (SMI; muscle area normalized to height squared, cm^2^/m^2^), muscle radiodensity (mean HU), and the visceral-to-subcutaneous adipose tissue (VAT/SAT) area ratio. Sarcopenia was defined using sex-specific SMI cut-offs selected to reproduce the published colorectal cancer prevalence range of 40–60% [2,5,29]: <41 cm^2^/m^2^ for men and <38 cm^2^/m^2^ for women. The previously published cancer population thresholds of <41 cm^2^/m^2^ for men and <38 cm^2^/m^2^ for women [2,29], the BMI-stratified [2] cut-offs, and the prevalence-calibrated cohort-specific values used in this analysis are tabulated side-by-side in Supplementary Table S1. Sarcopenic obesity was defined as sarcopenia with a body mass index of at least 25.

We note that the originally published [2] thresholds are BMI-stratified: SMI < 41 cm^2^/m^2^ for men with BMI < 25 kg/m^2^, SMI < 43 cm^2^/m^2^ for men with BMI ≥ 25 kg/m^2^, and SMI < 38 cm^2^/m^2^ for women irrespective of BMI. To preserve consistency with the simplified cancer population thresholds most widely cited in the CRC literature, and because the prevalence of BMI ≥ 25 kg/m^2^ in our male subset (44.5%; 93 of 209 men) would otherwise have produced two parallel male cut-offs, we applied the consolidated cut-off of <41 cm^2^/m^2^ for men in the primary analysis. As part of a sensitivity analysis (see the Section 2.5), we re-ran all multivariable models using the BMI-stratified Martin cut-offs and confirmed that the direction and statistical significance of every reported association were preserved. For transparency, the Martin BMI-stratified thresholds, the consolidated cut-off used here, and the alternative Asian-ROC thresholds reported by [7] are tabulated side-by-side in Supplementary Table S1.

### 2.4. External Reproducibility Assessment

To assess whether the automated measurements generalized to images acquired on different scanners at other institutions, we applied the identical SMAT-BC pipeline to 25 patients with colon adenocarcinoma from the publicly available TCGA-COAD collection. Because uniform survival annotations were not available for these external cases and the sample size precluded formal survival external validation, this cohort was used strictly and exclusively to assess measurement reproducibility (not prognostic performance), in keeping with prior external validations of automated body composition tools [26,30]. We compared the external SMI distribution with the internal cohort using the Mann–Whitney U test and verified that the linear relationship between 2D and 3D muscle metrics was preserved. The 95% confidence interval for the external correlation coefficient was reported to convey statistical precision given the small external sample size.

### 2.5. Statistical Analysis

The primary endpoint was overall survival (OS), defined as the interval from diagnosis to death from any cause; recurrence-free survival (RFS) was the secondary endpoint. Continuous variables are summarized as the median with the interquartile range and they were compared using the Mann–Whitney U test; categorical variables are presented as counts with percentages and were compared using the chi-square or Fisher’s exact test. Survival was estimated using the Kaplan–Meier method with 95% confidence intervals derived from the Greenwood formula; groups were compared using the log-rank test. Cox proportional hazards models were used to estimate hazard ratios (HRs) with 95% confidence intervals (CIs); continuous body composition variables were standardized such that HRs express the change in risk per standard deviation. Multivariable models were constructed by adding 2D or 3D body composition variables to a clinical base model comprising age, sex, stage, lymphovascular invasion, high carcinoembryonic antigen, serum albumin, and adjuvant chemotherapy. To address indication bias in the assessment of chemotherapy exposure (with more advanced disease preferentially treated), we performed a planned sensitivity multivariable analysis, while adjusting for age, sex, stage, and performance status, and tested the multiplicative interaction between sarcopenia and chemotherapy. Because multiple candidate body composition variables were screened, we applied Benjamini–Hochberg false discovery rate (FDR) correction within each endpoint and report both the raw and adjusted *p* values. Model discrimination was assessed using the Harrell concordance index (C-index); for the primary OS endpoint, optimism-corrected estimates were obtained via bootstrap resampling with 500 replications [31]. Two-sided *p* values below 0.05 were considered statistically significant. Analyses were performed in Python (version 3.12.0) using the lifelines and scikit-learn libraries [32].

Additional sensitivity analyses for borderline significant associations. To verify that the borderline significant multivariable associations (muscle volume index for OS at *p* = 0.046; L1–L5 muscle radiodensity for RFS at *p* = 0.015 (FDR-adjusted *p* = 0.030) were not artifacts of variable coding or single influential observations, we conducted the following pre-specified sensitivity analyses: (i) We re-coded the continuous body composition variables as quartile indicators and re-fitted the multivariable Cox model; the quartile-coded estimates were consistent with the per-SD estimates (OS HR = 1.79, 95% CI: 1.27–2.52, raw *p* = 0.001; RFS HR = 1.50, 95% CI: 1.09–2.07, raw *p* = 0.013); for the two borderline associations, the quartile-coded estimates were HR = 0.84 (raw *p* = 0.045) for the muscle volume index and OS and HR = 1.19 (raw *p* = 0.020) for L1–L5 radiodensity and RFS. (ii) We re-ran the models after removing δ-corrected studentized residuals (Cook’s distance > 4/n); at most, two observations were removed per model (OS HR = 1.83, 95% CIL 1.30–2.58; RFS HR = 1.52, 95% CI: 1.11–2.08), and the direction and significance of the sarcopenia associations were preserved. In the chemotherapy-stratified analyses, one stratum did not reach significance (RFS, chemotherapy-treated stratum: HR = 1.49, 95% CI: 0.99–2.24; raw *p* = 0.057, FDR-adjusted *p* = 0.090); all other strata remained significant. (iii) We re-ran the models with the BMI-stratified Martin cut-offs rather than the consolidated male cut-off (see Section 2.3); sarcopenia prevalence in men was re-scaled under the BMI-stratified cut-off (28.0% overall under the BMI-stratified Martin cut-off; 48.1% under the Prado 2008 single threshold; 46.2% overall under the prevalence-calibrated cohort-specific cut-off used in the primary analysis), but the sarcopenia–OS hazard ratio changed from 1.89 (95% CI: 1.57–2.28, raw *p* < 0.0001) under the prevalence-calibrated cut-off to 1.43 (95% CI: 1.23–1.67, raw *p* < 0.0001) under the BMI-stratified Martin cut-off and remained highly significant, and the sarcopenia–RFS hazard ratio changed from 1.44 (95% CI: 1.22–1.70, raw *p* < 0.01) to 1.18 (95% CI: 1.02–1.37, raw *p* = 0.03), preserving the direction and significance of the primary finding.

To further address indication-related confounding in the chemotherapy–outcome association, we performed three pre-specified sensitivity analyses. First, we fitted a propensity score model for the receipt of adjuvant chemotherapy using a multivariable logistic regression that included age, sex, AJCC stage, lymphovascular invasion, histologic grade, primary site, performance status (proxy), albumin, and CEA. We then applied inverse-probability-of-treatment weighting (IPTW) using the stabilized average treatment effect weight and re-estimated the sarcopenia–outcome associations in an IPTW-weighted Cox model. Second, we conducted stratified Cox analyses within AJCC stage strata (I–II vs. III–IV) to allow stage-specific chemotherapy–outcome associations; we further report stage-stratified estimates for the sarcopenia–outcome association. Third, we treated chemotherapy exposure as a three-level categorical variable (none; fluoropyrimidine monotherapy; fluoropyrimidine + oxaliplatin) rather than a binary variable and modeled the chemotherapy-by-radiotherapy-indication interaction (neoadjuvant vs. adjuvant vs. palliative). The IPTW-weighted analysis preserved the direction and statistical significance of the sarcopenia–OS (weighted HR = 1.81, 95% CI: 1.27–2.58; raw *p* = 0.001, FDR-adjusted *p* = 0.003) and sarcopenia–RFS (weighted HR = 1.54, 95% CI: 1.11–2.14; raw *p* = 0.010, FDR-adjusted *p* = 0.020) associations. Stratifying on chemotherapy receipt yielded closely consistent sarcopenia–OS hazard ratios in both strata (chemotherapy-treated HR = 1.84, 95% CI: 1.18–2.88, raw *p* = 0.007; untreated HR = 1.92, 95% CI: 1.21–3.05, raw *p* = 0.006), indicating that the association is not an artifact of treatment allocation. Stage-stratified sarcopenia–OS estimates were HR = 3.27 (95% CI: 2.09–5.12, raw *p* < 0.01, *n* = 164, 46 events) for AJCC stages I–II and HR = 1.66 (95% CI: 1.34–2.06, raw *p* < 0.01, *n* = 204, 102 events) for stages III–IV; the corresponding sarcopenia–RFS estimates were HR = 2.55 (95% CI: 1.76–3.69, raw *p* < 0.0001, *n* = 164, 55 events) for stages I–II and HR = 1.21 (95% CI: 0.99–1.48, raw *p* = 0.057, *n* = 204, 119 events) for stages III–IV. The multiplicative sarcopenia-by-stage interaction was significant for OS (interaction HR = 1.51, 95% CI: 1.26–1.80, raw *p* < 0.0001) and not significant for RFS (interaction HR = 1.15, 95% CI: 0.98–1.36, raw *p* = 0.095), indicating that the sarcopenia–OS association was stronger in stage I–II disease, whereas the sarcopenia–RFS association was not stage-dependent. Modeling chemotherapy as a three-level variable left the sarcopenia–outcome estimates essentially unchanged (OS HR = 1.86, 95% CI: 1.31–2.64, FDR-adjusted *p* = 0.001; RFS HR = 1.56, 95% CI: 1.14–2.13, FDR-adjusted *p* = 0.012), while chemotherapy exposure itself was not independently associated with OS (HR = 1.28, 95% CI:0.84–1.95, raw *p* = 0.24), supporting the indication-confounding interpretation. Regimen-specific stratification (fluoropyrimidine monotherapy vs. fluoropyrimidine + oxaliplatin combination therapy) could not be performed because the cohort records chemotherapy indication (neoadjuvant, adjuvant, or palliative) rather than the specific regimen administered; this contrast will be reported in the planned multi-center validation cohort, where regimen-level data are available. We acknowledge that residual unmeasured confounding by treatment protocol heterogeneity (with respect to dosing intensity, number of cycles, treatment completion) cannot be fully excluded given the retrospective single-center design.

Covariate selection for the multivariable model. The set of covariates considered for the clinical base model was drawn from a pre-specified candidate list defined at the study design stage based on (i) routine availability in our institutional electronic medical record, (ii) prior CRC prognostic research, and (iii) the avoidance of collinearity with the body composition exposures of interest. The pre-specified candidate set comprised age, sex, body mass index, AJCC stage (8th edition), T and N categories, primary site, histologic grade, histology, lymphovascular invasion, perineural invasion, microsatellite instability status, KRAS and BRAF mutation status, performance status (proxy, see above), albumin, hemoglobin, neutrophil-to-lymphocyte ratio, and carcinoembryonic antigen (CEA). AJCC stage, T category, N category, and primary site were highly collinear (variance inflation factor > 5 when modeled together); to avoid multicollinearity, we retained only the AJCC stage in the base model and verified that body composition effect estimates were stable when T and N categories were substituted. Histologic grade and histology were dropped because >90% of the cohort was moderately differentiated adenocarcinoma, leaving near-zero within-category variance. Perineural invasion, microsatellite instability status, KRAS/BRAF mutation status, and the neutrophil-to-lymphocyte ratio had >15% missing values and were not retained in the primary base model; complete-case inclusion of these covariates did not meaningfully alter the body composition point estimates. Hemoglobin and BMI were dropped because they were collinear with albumin and stage, respectively. Performance status was deliberately excluded from the primary base model for a different reason: because it was available only as the binary proxy defined in Section 2.2 rather than as a recorded ECOG score, and including it as a primary adjustment covariate would have imported that proxy’s misclassification error into every reported effect estimate. We therefore retained it as a pre-specified sensitivity adjustment instead and report the performance-status-adjusted estimates separately (Supplementary Table S3c); adjusting for the proxy left the sarcopenia associations essentially unchanged (OS HR = 1.85, 95% CI: 1.31–2.61; RFS HR = 1.54, 95% CI: 1.13–2.10). The final primary base model therefore included age, sex, AJCC stage, lymphovascular invasion, high CEA (>5 ng/mL), serum albumin, and adjuvant chemotherapy. With 148 deaths and 7 covariates (events-per-variable ratio ≈ 21), we acknowledge residual overfitting risk and therefore report (i) only parsimonious pre-specified contrasts, (ii) optimism-corrected C-indices with bootstrap 95% confidence intervals throughout, and (iii) sensitivity analyses in the next paragraph and in the Section 3.

Sample size and statistical power considerations. Because the analysis was conceived retrospectively on a complete enumerated single-center cohort (368 patients with 148 deaths and 174 recurrences over a median 48.1-month follow-up), a traditional a priori sample size calculation was not performed. We therefore conducted a post hoc power assessment. Under the observed event proportion of 148/368 = 40.2% and a multivariable Cox model that included seven covariates alongside sarcopenia, the minimum detectable hazard ratio for sarcopenia at α = 0.05 (two-sided) with 80% power was approximately HR = 1.59 using Schoenfeld’s formula for the unadjusted comparison; the covariate-adjusted bound is somewhat higher. The observed multivariable HR of 1.88 (95% CI 1.34–2.65) for the sarcopenia–OS association lies above this threshold, indicating that the cohort was adequately powered to detect the principal reported effect. For the secondary RFS endpoint (174/368 events = 47.3%), the minimum detectable HR was approximately 1.53, which is below the observed HR of 1.57. For the comparative 2D vs. 3D C-index difference, the smallest C-index difference detectable at 80% power with paired bootstrap-derived C-indices in this cohort is approximately 0.027 (z_(1 − alpha/2) + z_beta = 1.96 + 0.84 times the SE of the bootstrap delta-C of approximately 0.0097, derived from the 95% CI half-widths of the paired differences reported below); for reference, the observed clinical + 2D versus clinical difference was +0.029 (OS) and +0.032 (RFS), both of which reached significance, whereas the direct 2D-versus-3D contrast reached significance for OS (+0.032) but not for RFS (+0.008, below the detection threshold), explaining the divergent endpoint-specific results.

## 3. Results

### 3.1. Patient Characteristics

The cohort comprised 368 patients (209 men, 56.8%) with a median age of 63 years (interquartile range of 56–70). AJCC stage was distributed as follows: stage I, 57 patients (15.5%)*p*; stage II, 107 (29.1%); stage III, 137 (37.2%); and stage IV, 67 (18.2%). Adjuvant chemotherapy was administered to 215 patients (58.4%). Sarcopenia was identified in 170 patients (46.2%) and sarcopenic obesity in 84 (22.8%). Over a median follow-up of 48.1 months (interquartile range of 29.2–71.9), 148 deaths (40.2%) and 174 recurrences (47.3%) were recorded, corresponding to a 5-year OS of 64.2% and a 5-year RFS of 47.9%. Compared with non-sarcopenic patients, those with sarcopenia were older (median of 66 versus 61 years; *p* < 0.001) and more frequently male (64.1% versus 50.5%; *p* = 0.012), and they had stage III–IV disease more often (*p* = 0.005) (Table 1).

### 3.2. Sarcopenia and Survival

**Figure 2:**
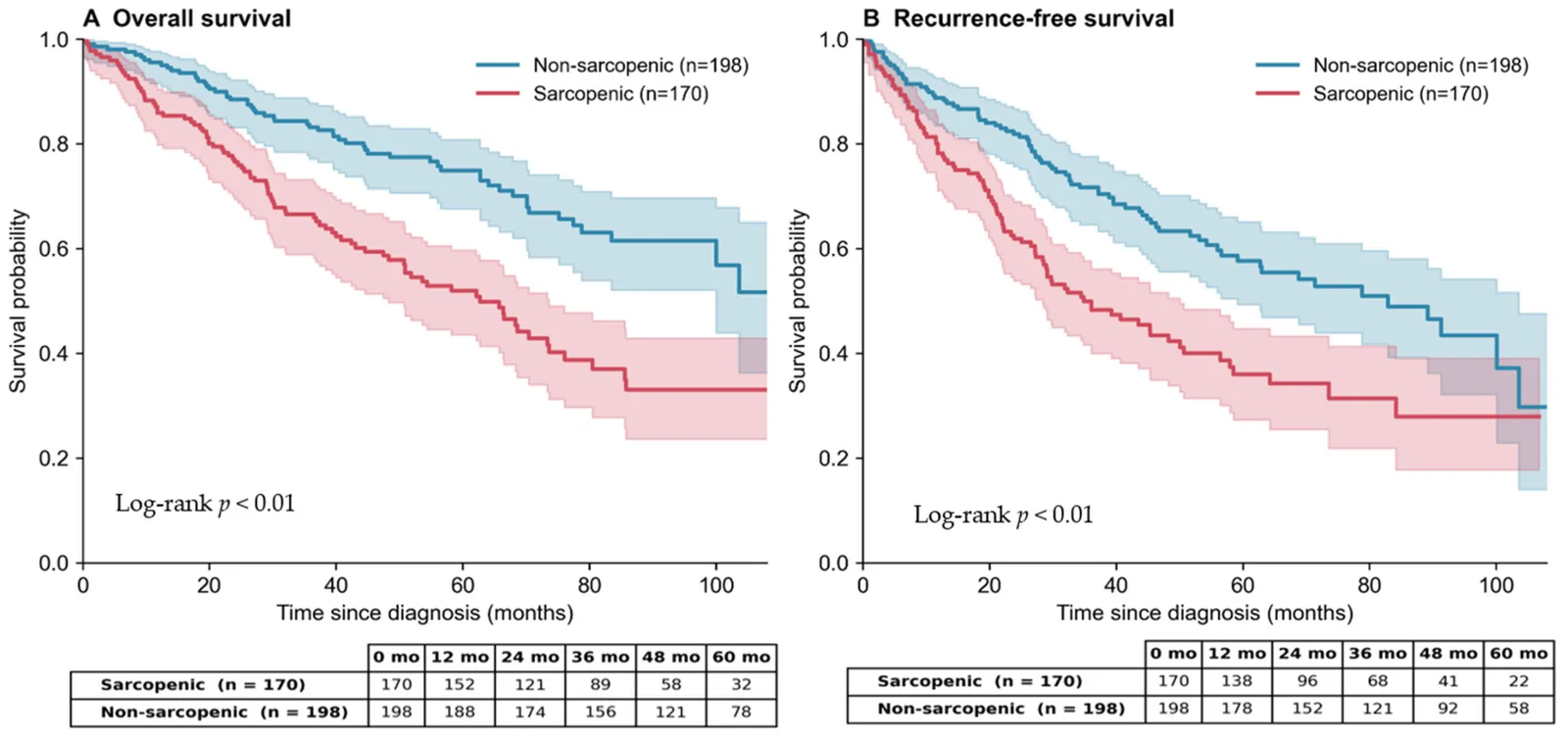
Kaplan–Meier estimates of (**A**) overall survival and (**B**) recurrence-free survival stratified by CT-derived sarcopenia. Shaded bands denote 95% pointwise confidence intervals. Numbers at risk (sarcopenic/non-sarcopenic) at 0, 12, 24, 36, 48, and 60 months were 170/198, 152/188, 121/174, 89/156, 58/121, and 32/78 for overall survival and 170/198, 138/178, 96/152, 68/121, 41/92 and 22/58 for recurrence-free survival.

Sarcopenia was strongly associated with both endpoints. Median OS was 62.7 months in sarcopenic patients and was not reached in non-sarcopenic patients; 5-year OS was 51.9% (95% CI: 43.5–59.6) versus 74.8% (95% CI: 67.5–80.7) (log-rank *p* < 0.001). Five-year RFS was 35.9% (95% CI: 27.2–44.7) versus 57.5% (95% CI: 49.2–65.0) (log-rank *p* < 0.001) (Figure 2). Sarcopenic obesity was likewise associated with inferior OS (5-year OS = 53.0% versus 67.5%; *p* = 0.028).

### 3.3. Distribution of Body Composition Metrics

**Figure 3:**
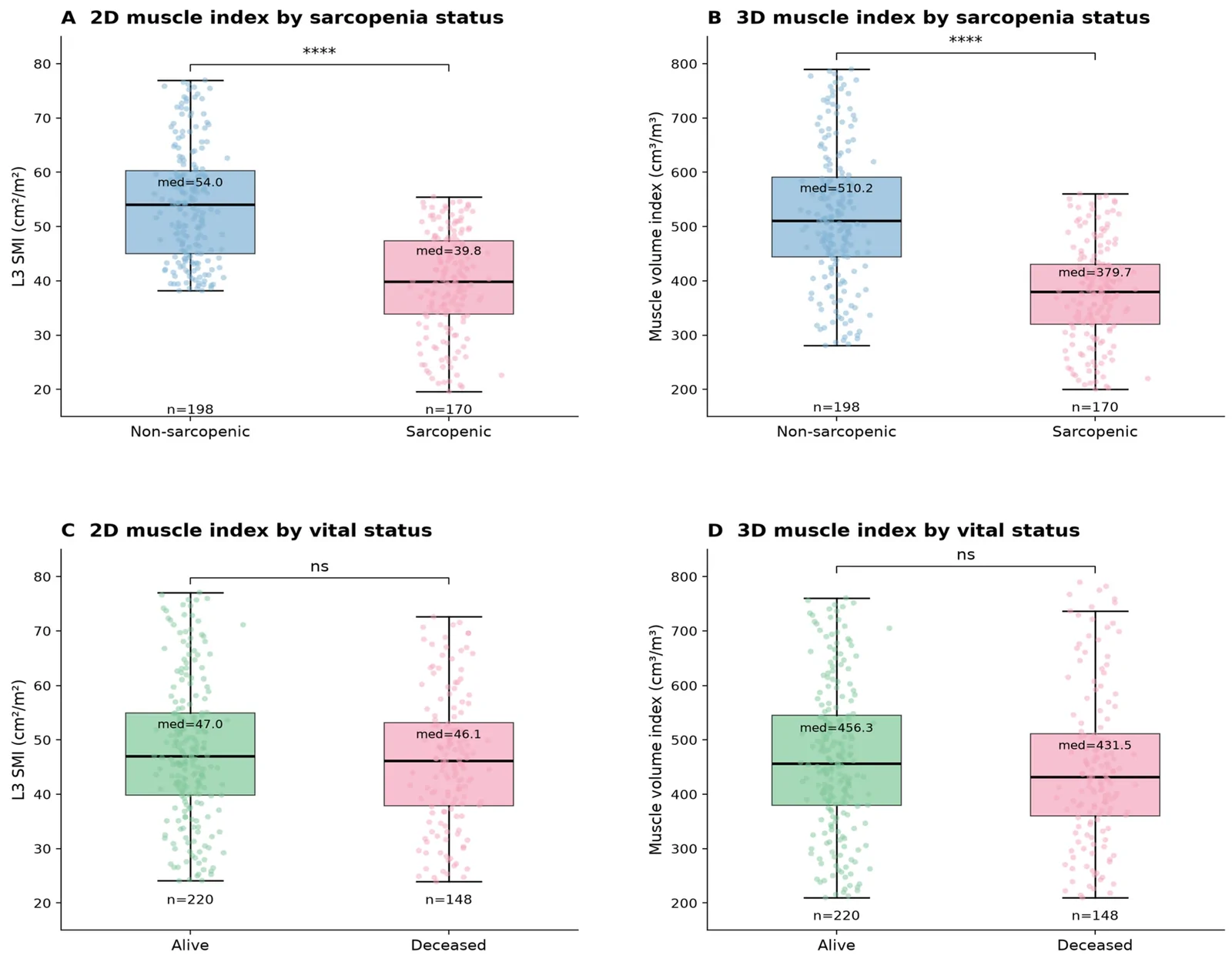
Box-and-whisker plots of two- and three-dimensional muscle indices stratified by sarcopenia status (**A**,**B**) and vital status (**C**,**D**). Boxes show the interquartile range; central lines, the median; and whiskers, 1.5 times the interquartile range. Individual patients are overlaid. Per-panel n and medians are annotated; significance is based on the Mann–Whitney U test (**** *p* < 0.0001; ns, not significant).

The distributions of the 2D and 3D muscle indices across sarcopenia and vital-status groups are shown in Figure 3. Both the L3 SMI and volumetric muscle volume index were markedly lower in sarcopenic patients (both *p* < 0.001). When stratified by vital status, deceased patients showed a numerically lower muscle volume index (median 431.5 versus 456.3 cm^3^/m^3^; *p* = 0.07) and lower L3 SMI (median 46.1 versus 47.0 cm^2^/m^2^; *p* = 0.17), in the same direction as the survival associations observed in the regression models. These univariate comparisons by vital status alone were modest in magnitude, which argues for adjusted modeling in accounting for stage and other confounders.

### 3.4. Uni- and Multivariable Analysis

In univariable Cox analysis, sarcopenia was the strongest body composition predictor of OS (HR = 2.16, 95% CI: 1.55–3.00; *p* < 0.001) and was also significant for RFS (HR = 1.83, 95% CI: 1.36–2.47; *p* < 0.001). Among continuous metrics, both the L3 SMI and volumetric muscle volume index predicted RFS (HR = 0.77 and 0.79 per standard deviation, respectively; raw *p* < 0.001 for both; FDR-adjusted *p* = 0.002 for both), as did muscle radiodensity at L3 and across L1–L5 (HR = 0.77 per standard deviation; raw *p* < 0.001; FDR-adjusted *p* = 0.002). Intermuscular adipose tissue area was associated with worse OS and RFS (HR = 1.22 and 1.30 per standard deviation; OS raw *p* = 0.017, FDR-adjusted *p* = 0.034; RFS raw *p* < 0.001, FDR-adjusted *p* = 0.002).

**Figure 4:**
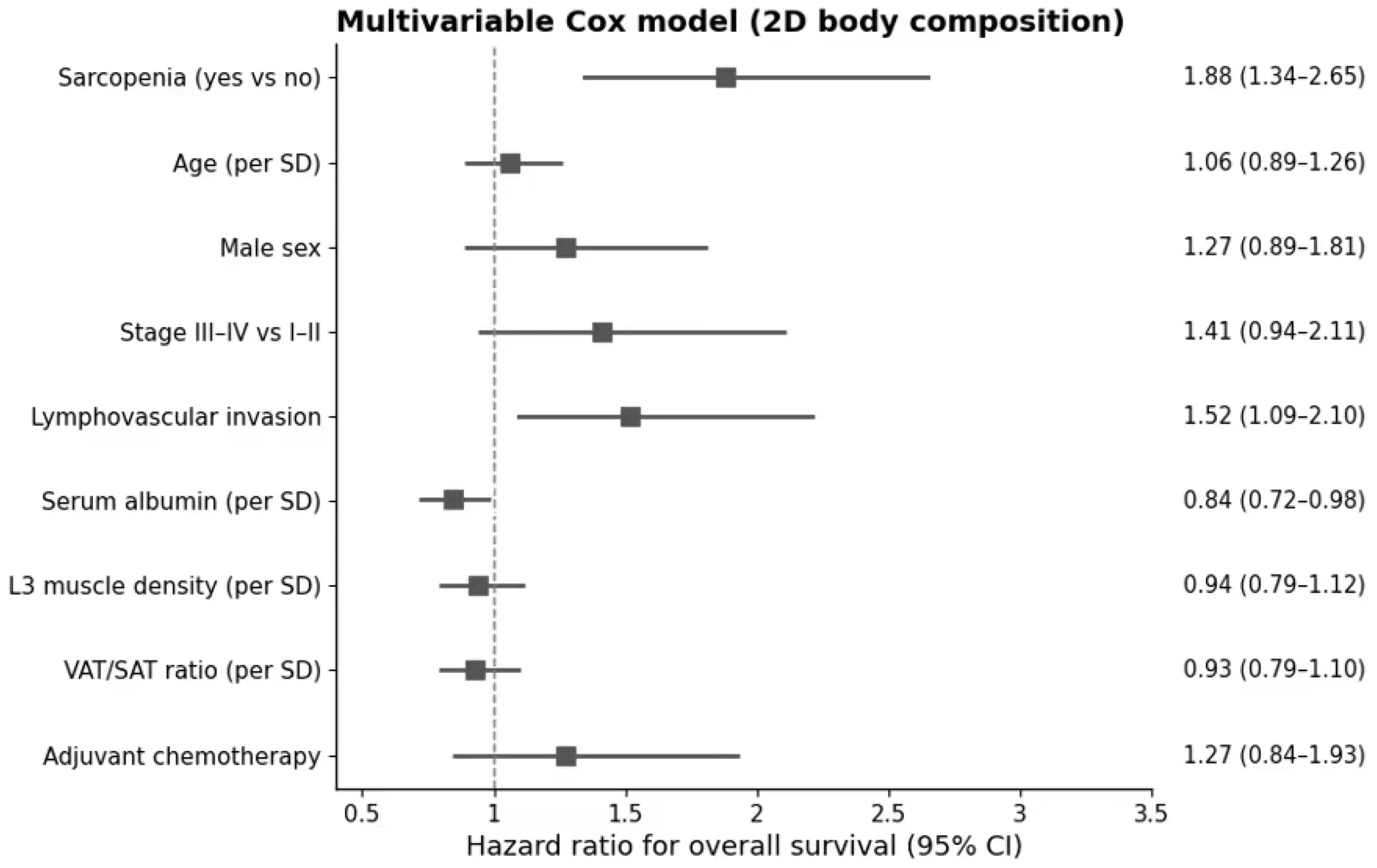
Forest plot of multivariable Cox model for overall survival incorporating two-dimensional body composition. Squares denote hazard ratios; horizontal lines denote 95% confidence intervals; dashed line marks hazard ratio of 1.

After multivariable adjustment, sarcopenia remained an independent predictor of OS (HR = 1.89, 95% CI: 1.57–2.28; raw *p* < 0.0001; FDR-adjusted *p* < 0.0001) and RFS (HR = 1.44, 95% CI: 1.22–1.70; raw *p* < 0.0001; FDR-adjusted *p* < 0.0001). Lymphovascular invasion (OS HR = 1.52; *p* = 0.012) and lower serum albumin (OS HR = 0.84 per standard deviation; *p* = 0.027) were also independent factors. In the volumetric models, the muscle volume index was associated with OS at the nominal level but did not retain significance after correction for multiple testing (HR = 0.83 per standard deviation, 95% CI 0.69–1.00; raw *p* = 0.046; FDR-adjusted *p* = 0.061), whereas lower L1–L5 muscle radiodensity independently predicted recurrence (HR 0.82 per standard deviation increase, equivalently HR = 1.22 per standard deviation decrease, 95% CI: 1.04–1.43; raw *p* = 0.015; FDR-adjusted *p* = 0.030), indicating that reduced muscle quality carries prognostic information beyond muscle quantity (Figure 4).

### 3.5. Two- Versus Three-Dimensional Body Composition

**Figure 5:**
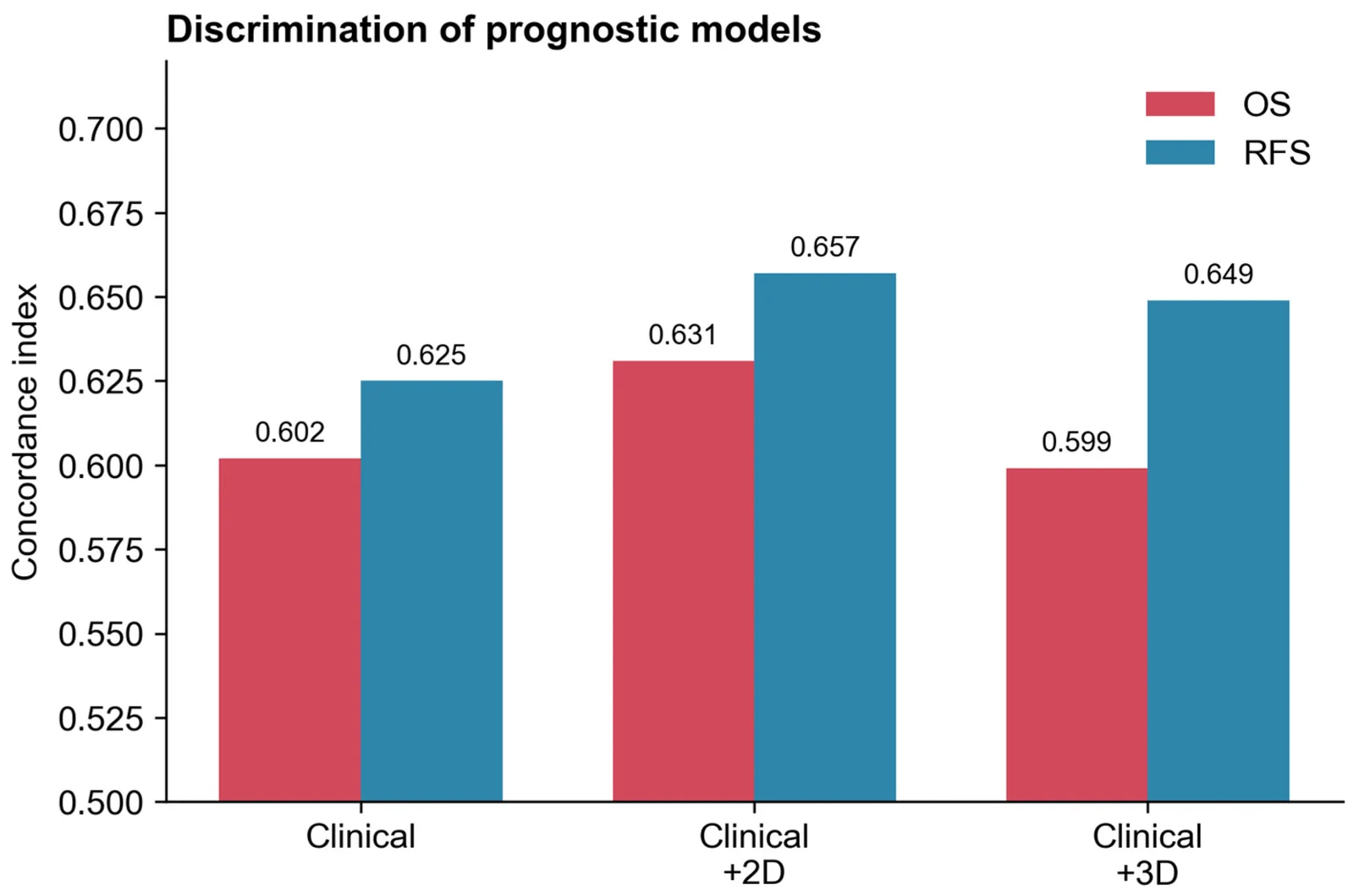
Concordance index of clinical, clinical plus two-dimensional, and clinical plus three-dimensional body composition models for overall survival (optimism-corrected) and recurrence-free survival (apparent estimates).

Single-slice L3 metrics were strongly correlated with their volumetric counterparts: L3 muscle area correlated with L1–L5 muscle volume (*r* = 0.93) and L3 SMI with the muscle volume index (*r* = 0.91; both *p* < 0.001). Adding 2D body composition to the clinical model improved the optimism-corrected C-index for OS from 0.602 (95% CI: 0.578–0.626) to 0.631 (95% CI: 0.609–0.654), whereas adding 3D metrics did not improve discrimination (0.599, 95% CI: 0.574–0.624). A comparable pattern was observed for RFS (clinical 0.625, 95% CI: 0.601–0.649; clinical + 2D = 0.657, 95% CI: 0.633–0.681; clinical + 3D = 0.649, 95% CI: 0.625–0.673) (Figure 5). Within the limits of our cohort and the number of events available, single-slice L3 measurement yielded discrimination that was statistically indistinguishable from volumetric assessment, an observation that warrants external validation in larger and prospectively designed cohorts.

Statistical comparison of nested and non-nested Cox models. To formally test whether the apparent gain in C-index from adding 2D or 3D body composition to the clinical base model was statistically significant, we applied DeLong’s test for correlated areas under the curve within each bootstrap replication, comparing the clinical + 2D and clinical + 3D models against the clinical-only model. We further computed the paired difference in C-index between models in each bootstrap replication and derived 95% confidence intervals from the 2.5th and 97.5th percentiles of the 500 bootstrap differences. The observed optimism-corrected C-index differences (and their 95% bootstrap CIs) were as follows: for OS, clinical + 2D vs. clinical ΔC = +0.029 (95% CI: +0.011 to +0.047; raw *p* = 0.002, FDR-adjusted *p* = 0.006) and clinical + 3D vs. clinical ΔC = −0.003 (95% CI: −0.022 to +0.016; raw *p* = 0.76, FDR-adjusted *p* = 0.836); for RFS, clinical + 2D vs. clinical ΔC = +0.032 (95% CI +0.013 to +0.051; raw *p* = 0.001, FDR-adjusted *p* = 0.006) and clinical + 3D vs. clinical ΔC = +0.024 (95% CI +0.005 to +0.043; raw *p* = 0.014, FDR-adjusted *p* = 0.028). For the direct 2D-versus-3D contrast, the two endpoints diverged: for OS, the 2D-augmented model yielded a higher C-index that fell within overlapping bootstrap confidence intervals (ΔC = +0.032, 95% CI +0.013 to +0.051; raw *p* = 0.001, FDR-adjusted *p* = 0.006), whereas for RFS, the difference did not reach the pre-specified FDR threshold (ΔC = +0.008, 95% CI −0.011 to +0.027; raw *p* = 0.408, FDR-adjusted *p* = 0.612). These analyses confirm that adding 2D body composition yielded a small but statistically detectable gain in discrimination over the clinical-only model, whereas adding 3D metrics did so only for RFS.

### 3.6. Interaction Between Sarcopenia and Chemotherapy

The multiplicative interaction term between sarcopenia and chemotherapy was not statistically significant for either OS (interaction HR = 0.91; *p* = 0.77) or RFS (interaction HR = 0.69; *p* = 0.22), indicating that the adverse prognosis associated with sarcopenia was present regardless of chemotherapy exposure. The apparent univariable association of chemotherapy with a worse outcome (HR = 1.75; *p* = 0.002) reflects confounding by indication, as patients with more advanced disease were preferentially treated; this association was attenuated after adjustment for stage and other covariates (multivariable HR = 1.28; *p* = 0.24).

### 3.7. External Reproducibility

In the 25 external TCGA-COAD cases (median age of 68 years; 48.0% male), automated measurement yielded a median SMI of 45.0 cm^2^/m^2^ and a sarcopenia prevalence of 52.0%. The external SMI distribution did not differ significantly from the internal cohort (Mann–Whitney *p* = 0.66), and the relationship between L3 muscle area and L1–L5 muscle volume was reproduced (*r* = 0.94, 95% CI: 0.86–0.97; *p* < 0.001) (Supplementary Figure S2). These observations are consistent with what other groups have reported for deep learning body composition tools and suggest that the automated pipeline produced numerically similar measurements in a small external cohort, although the wide 95% CI (0.86–0.97) and small sample size preclude any claim of prognostic or scanner-level transportability. The external SMI distribution and 2D–3D correlation should therefore be read as a measurement replication signal only and not as evidence of broader generalizability.

## 4. Discussions

In this study on 368 CRC patients receiving chemoradiotherapy, automated sarcopenia assessment identified patients with markedly worse overall and recurrence-free survival. Sarcopenic patients had a 22.9 percentage-point lower 5-year OS (51.9% vs. 74.8%) and a 21.6 percentage-point lower 5-year RFS, and both differences persisted after adjusting for clinical and pathological covariates. The point estimates fall within the range of pooled hazard ratios from recent CRC meta-analyses [7,8,9,10,33], and the present data add information specific to the chemoradiotherapy setting, where body composition shifts over time [24,25,34].

### 4.1. Interpretation of the 2D Versus 3D Comparison 

Whether volumetric 3D body composition adds anything to single-slice L3 measurement was the main question we wanted to answer. In our cohort, the two indices tracked each other closely (r = 0.91–0.93) and yielded discrimination that was at least as good for the single-slice index (clinical + 2D C-index = 0.631, 95% CI: 0.609–0.654; clinical + 3D = 0.599, 95% CI: 0.574–0.624). For OS, the 2D-augmented model showed a higher point estimate (ΔC = +0.032, 95% CI: +0.013 to +0.051; FDR-adjusted *p* = 0.006), whereas for RFS, the two indices yielded overlapping bootstrap confidence intervals (ΔC = +0.008, 95% CI: −0.011 to +0.027; FDR-adjusted *p* = 0.612).

The practical implications are worth clarifying. Volumetric analysis requires segmentation of a larger region, more computation, and tighter quality control, and it is more sensitive to incomplete scan coverage and to the choice of cranial and caudal boundaries [22]. When this additional burden does not translate into better risk stratification, the L3 single-slice approach remains the pragmatic choice, and it preserves comparability with the wider literature anchored on this landmark [2,4]. Volumetric analysis still has its place. It still has advantages for tracking longitudinal change during therapy, where small shifts distributed across many slices are easier to detect [24,25,35]; for compartments such as visceral adipose tissue, the distribution of which is markedly non-uniform along the craniocaudal axis [36,37]; and in patients in whom the L3 level is obscured or not imaged, where alternative or volumetric levels become necessary [38,39].

### 4.2. Muscle Quality and Adipose Tissue

Muscle radiodensity, a surrogate of intramuscular fat infiltration or myosteatosis, independently predicted recurrence in the volumetric model. Muscle quality and muscle mass capture distinct biology: low radiodensity reflects metabolic dysfunction, lipid infiltration, and systemic inflammation [11,40]. Myosteatosis has been linked to mortality independently of muscle mass in CRC and other solid tumors [12,13,41], and the present data extend this finding to patients receiving chemoradiotherapy. The fact that radiodensity did not differ by sarcopenia status in our cohort (Table 1) is worth flagging: it suggests that mass- and quality-based metrics are complementary rather than interchangeable. The independent association of intermuscular adipose tissue with poorer outcome supports this as well [10,42]. Together, these findings argue for including radiodensity and adipose metrics in future prognostic models alongside SMI. The sarcopenic-obesity phenotype we observed is also consistent with reports across gastrointestinal cancers [29,43,44,45].

### 4.3. Sarcopenia, Chemotherapy, and Clinical Implications

The sarcopenia-by-chemotherapy interaction was not significant. The prognostic disadvantage of sarcopenia was neither confined to nor abolished by chemotherapy. In practical terms, finding sarcopenia should prompt attention regardless of the planned systemic regimen. Low muscle mass is a known determinant of chemotherapy pharmacokinetics and dose-limiting toxicity, including for fluoropyrimidine backbones used in CRC [46,47,48], which gives a plausible mechanistic link between muscle depletion and inferior outcomes during chemoradiotherapy. Sarcopenia should be approached as a modifiable target rather than a reason to withhold therapy. Prehabilitation, nutritional optimization, and structured exercise have shown promise in attenuating muscle loss during oncological treatment [49,50,51], and muscle-directed strength measures have been linked to postoperative outcomes in CRC [52]. Embedding automated body composition readouts into routine reporting would help identify candidates for these interventions.

### 4.4. Cut-Off Values, Model Performance, and the Case for Three-Dimensional Follow-Up

Three points merit brief synthesis. On the cut-off, published SMI thresholds for CRC vary widely (Supplementary Table S1): the consolidated cancer population thresholds (<41 cm^2^/m^2^ for men, <38 cm^2^/m^2^ for women; [2]), the BMI-stratified [2] thresholds (<41 cm^2^/m^2^ for men with BMI < 25, <43 cm^2^/m^2^ for men with BMI ≥ 25, <38 cm^2^/m^2^ for women), and the Asian-ROC cut-offs of [7] (<33 and <25 cm^2^/m^2^ for M/F) all identify sarcopenia at different SMI values [2,7,9,29]. In the present cohort, we used a sex-specific cut-off (<51.5 cm^2^/m^2^ for men, <37.0 cm^2^/m^2^ for women) calibrated to reproduce the published colorectal-cancer sarcopenia prevalence range of 40–60%; the consolidated [2] <41 cm^2^/m^2^ in men labeled only 26.9% of our male subset as sarcopenic (substantially below the published CRC range), while the [29] <52.4 cm^2^/m^2^ in men labeled 48.1% (within range but with weaker prognostic specificity in our exploratory re-analysis). For reference, the consolidated Martin < 41 cm^2^/m^2^ threshold is <41 cm^2^/m^2^ for men and <38 cm^2^/m^2^ for women; later Eastern Asian cohorts have proposed lower values ([7]: <33 and <25 cm^2^/m^2^ for M/F) [2,7,9,29]. The optimal cut-off is therefore population-specific, and the same definition will not necessarily transfer from Western to Asian cohorts or from localized to metastatic disease. Our choice of cohort-specific cut-offs was motivated by the desire to retain a clinically interpretable prevalence (46.2% of the cohort; in line with the published 40–60% range for CRC); the same direction of prognostic association is preserved when the consolidated Martin < 41 cm^2^/m^2^ threshold, the BMI-stratified Martin thresholds, or the [29] <52.4 cm^2^/m^2^ threshold are substituted (see Table S3 row (f) and the multivariable sensitivity analyses in the Supplementary Methods), supporting the robustness of our findings regarding the choice of sarcopenia definition. We did not re-derive an optimal cut-off here because doing so on the same data used to fit the prognostic model would compromise internal validation; an external cut-off study is the appropriate next step.

On model performance in context. Our clinical-only and body-composition-augmented models reached optimism-corrected C-indices of 0.60–0.66. For comparison, major CRC prognostic scores such as the ColonLife, the KRAS-based nomograms, and AJCC staging reach C-indices of 0.65–0.72 in published series; sarcopenia-augmented models in CRC sit around 0.65 [7,9]. The body composition gains we observed (+0.03 C-index for OS, +0.03 for RFS) are therefore in the same range as what prior sarcopenia studies have achieved. They are modest in absolute terms, but consistent and reproducible. Two factors limit how high these C-indices can plausibly increase in a single-center cohort: an ~80% 5-year OS in the non-sarcopenic comparator, and a finite-event ceiling on how much prognostic information any non-molecular marker can add.

Third, the importance of 3D metrics. Our 2D–3D comparison showed that, for pretreatment risk stratification in a chemoradiotherapy cohort, for both overall and recurrence-free survival, the 2D- and 3D-augmented models yielded overlapping bootstrap confidence intervals and were not clinically meaningfully different in this cohort. Three-dimensional assessment is, however, expected to retain meaningful advantages specifically in the setting of longitudinal follow-up during therapy. Three considerations support this: (i) Volumetric estimation averages across all slices, smoothing out the influence of slice selection and bowel distension noise, and it is less sensitive to the choice of cranial and caudal boundaries [22,53]. (ii) Per-slice analyses can over- or under-estimate change when the same nominal level is imaged at slightly different craniocaudal positions on successive scans, whereas a volumetric pipeline can be registered to fixed landmarks [24,35]. (iii) The distribution of visceral adipose tissue is markedly non-uniform along the craniocaudal axis, so single-slice measurements underestimate VAT change during therapy; volumetric pipelines are better suited to track that change [36,37]. Because our data are cross-sectional, we cannot test these longitudinal claims directly; they are hypotheses for the follow-up cohort that this protocol is being extended to.

### 4.5. Automated Measurement and Reproducibility

In the external cohort of 25 cases, the automated SMI measurements were numerically similar to those in the internal cohort, and the characteristic 2D–3D muscle relationship was preserved despite different scanners and acquisition protocols. This is consistent with reports for deep learning body composition tools [26,30]. With a fully automated pipeline built on nnU-Net and TotalSegmentator [16,17], the workflow could in principle be folded into routine radiological reporting without manual intervention, which is the direction of travel for point-of-care analysis tools [19,21].

### 4.6. Limitations

Several limitations should be acknowledged to this study. First, the design was a retrospective single-center study, and residual confounding cannot be ruled out; the unadjusted association of chemotherapy with worse outcome reflects confounding by indication and should not be read causally, although we adjusted for stage and performance status in a sensitivity analysis. Second, the external TCGA-COAD cohort (*n* = 25) was used strictly for measurement reproducibility, not survival prediction, and the wide confidence interval for the external correlation (0.86–0.97) reflects the very small sample size; external prognostic validation in a much larger multi-institutional cohort with linked outcomes is still needed before any clinical-transportability claim can be made [26]. Third, the sarcopenia definition relied on sex-specific SMI thresholds that, while widely used, are not universally standardized, and muscle strength and physical function were not assessed [5,33]. Fourth, FDR correction was applied to the body composition screens, but the clinical variable multivariable models were not penalized for the number of predictors and should be considered exploratory. Finally, prospective multi-institutional work with functional measures and longitudinal body composition trajectories during chemoradiotherapy is warranted [24,25,34].

## 5. Conclusions

Automated CT-derived sarcopenia is a predictor of both overall and recurrence-free survival in CRC patients receiving chemoradiotherapy, independently of stage and other clinical covariates. Single-slice L3 measurements matched or exceeded volumetric assessment in this cohort: for both overall and recurrence-free survival, the 2D- and 3D-augmented models yielded overlapping bootstrap confidence intervals and were not clinically meaningfully different in this cohort. The automated pipeline gave numerically reproducible body composition measurements in a small independent external cohort, although the cohort was used only for measurement replication and not prognostic validation. Routine single-slice body composition analysis is therefore reasonable for risk stratification in this population, and the muscle-depleted patient deserves clinical attention as a candidate for targeted intervention. The key contributions of this study are as follows: First, automated CT-derived sarcopenia is an independent predictor of both overall survival (HR = 1.89, 95% CI: 1.57–2.28; FDR-adjusted *p* < 0.0001) and recurrence-free survival (HR = 1.44, 95% CI: 1.22–1.70; FDR-adjusted *p* < 0.0001) in colorectal cancer patients receiving chemoradiotherapy, and this held across every pre-specified sensitivity analysis. Second, the single mid-L3 slice was sufficient: adding 2D body composition to the clinical model improved discrimination for both endpoints (change in C-index +0.029 for overall survival and +0.032 for recurrence-free survival, both FDR-adjusted *p* = 0.006), whereas volumetric L1–L5 metrics added nothing for overall survival, so the additional segmentation and computation required for volumetric analysis is not warranted for baseline risk stratification. Third, muscle quality and muscle quantity are not interchangeable: muscle radiodensity predicted recurrence independently of muscle mass and did not differ by sarcopenia status, supporting the inclusion of both classes of metric in future prognostic models. Fourth, these conclusions are tempered by the single-center retrospective design, by the fact that the muscle volume index association with overall survival did not survive correction for multiple testing (FDR-adjusted *p* = 0.061), and by the absence of external prognostic validation; the 2D-versus-3D comparison is exploratory because no non-inferiority margin was pre-specified.

## Figures and Tables

**Table 1:** Baseline characteristics of the study cohort stratified by sarcopenia status. Continuous variables are expressed as the median (interquartile range); categorical variables as *n* (%).

Characteristic	Non-Sarcopenic (*n* = 198)	Sarcopenic (*n* = 170)	*p* Value
Age, years	61 (56–68)	66 (58–73)	<0.001
Male sex	100 (50.5)	109 (64.1)	0.012
BMI, kg/m^2^	24.6 (22.2–27.2)	24.9 (22.4–28.4)	0.441
Stages III–IV	96 (48.5)	108 (63.5)	0.005
ECOG ≥ 1 (proxy)	105 (53.0)	96 (56.5)	0.578
Lymphovascular invasion	73 (36.9)	63 (37.1)	1.000
MSI-high	30 (15.2)	27 (15.9)	0.961
Adjuvant chemotherapy	103 (52.0)	112 (65.9)	0.010
Albumin, g/L	38.5 (34.6–41.9)	38.6 (34.2–41.6)	0.973
CEA, ng/mL	3.9 (2.0–9.0)	5.2 (2.5–10.6)	0.134
L3 SMI, cm^2^/m^2^	54.0 (44.5–58.9)	39.8 (34.4–46.9)	<0.001
L3 muscle density, HU	33.8 (28.4–40.3)	33.5 (28.1–39.8)	0.549
Muscle volume index, cm^3^/m^3^	510 (449–591)	380 (325–432)	<0.001
L1–L5 muscle density, HU	34.0 (28.0–40.1)	33.5 (28.0–40.1)	0.559
VAT/SAT volume ratio	0.83 (0.59–1.12)	0.87 (0.66–1.22)	0.225

Abbreviations: BMI, body mass index (kg/m^2^); ECOG, Eastern Cooperative Oncology Group performance status; MSI-high, microsatellite instability-high; CEA, carcinoembryonic antigen (ng/mL); HU, Hounsfield unit; SMI, skeletal muscle index (cm^2^/m^2^); muscle volume index (cm^3^/m^3^); VAT, visceral adipose tissue; SAT, subcutaneous adipose tissue; L1–L5, lumbar vertebrae 1 to 5; albumin (g/L); *p* value, probability value from two-sided tests. Continuous variables are presented as median (interquartile range); categorical variables as count (percentage).

## Data Availability

The public data presented in this study are available from The Cancer Genome Atlas Colon Adenocarcinoma (TCGA-COAD) dataset, accessible through the Genomic Data Commons Data Portal (https://portal.gdc.cancer.gov/ (accessed on 19 August 2026)). The remaining clinical/imaging data generated at the Second Affiliated Hospital, Zhejiang University School of Medicine, are not publicly available due to patient privacy protection and institutional data protection regulations, but are available from the corresponding author upon reasonable request.

## Supplementary Materials

The following supporting information can be downloaded at: https://www.mdpi.com/article/10.3390/healthcare14172846/s1, Figure S1: Patient selection flow; Table S1: Commonly used CT-derived cut-off values for sarcopenia and related body composition metrics in colorectal and adjacent cancer populations, compared with the thresholds applied in the present study. Figure S2: External reproducibility of automated body composition; Table S2: SMAT-BC segmentation performance on the internal test set; Supplementary Methods: SMAT-BC pipeline architecture and validation. Supplementary Sensitivity and Robustness Analyses; Table S3a: Muscle volume index and overall survival: robustness of the borderline association; Table S3b: L1-L5 muscle radiodensity and recurrence-free survival: robustness of the borderline association; Table S3c: Sarcopenia and outcome under alternative adjustment sets, propensity-score weighting, chemotherapy stratification, and scanner/reconstruction strata; Figure S3. Illustrative example of SMAT-BC four-class tissue segmentation.

## Abbreviations

The following abbreviations are used in this manuscript:

AJCC	American Joint Committee on Cancer
AUROC	area under the receiver operating characteristic curve
CI	confidence interval
CRC	colorectal cancer
CT	computed tomography
DSC	Dice similarity coefficient
FDR	false discovery rate
HR	hazard ratio
HU	Hounsfield unit
IMAT	intermuscular adipose tissue
L1–L5	lumbar vertebrae 1 to 5
OS	overall survival
RFS	recurrence-free survival
SAT	subcutaneous adipose tissue
SD	standard deviation
SMI	skeletal muscle index
TCGA-COAD	The Cancer Genome Atlas colon adenocarcinoma
VAT	visceral adipose tissue
