# Supplementary material for: Computed Tomography-Derived Sarcopenia and Two- Versus Three-Dimensional Body Composition for Prognostication in Colorectal Cancer Patients Receiving Chemoradiotherapy: An Automated Deep Learning Segmentation Study with External Reproducibility Assessment
Source: Healthcare (Basel). 2026 Sep 4;14(17):2846. doi: 10.3390/healthcare14172846 (PMC13565101; doi:10.3390/healthcare14172846)
Supplement: Supplementary file 1 [file healthcare-14-02846-s001.zip › healthcare-4464145-supplementary.pdf]

Computed Tomography-Derived Sarcopenia and Two- Versus Three-Dimensional Body Composition for Prognostication in Colorectal Cancer Patients Receiving Chemoradiotherapy: An Automated Deep Learning Segmentation Study with External Reproducibility Assessment

Da Wang [1,2,*,†], Jiaping Sui [1,†], Jiaqi Chen [3], Lina Chen [4], Qi Yang [5], Yanting Wang [6,7] and Shuangxiang Lin [8,*]

Supplementary Materials

The following supporting information is available online. Figure S1: Patient selection flow. Figure S2: External 2D-3D body-composition reproducibility. Table S1: Commonly used CT-derived cut-off values for sarcopenia and related body composition metrics in colorectal and adjacent cancer populations. Table S2: SMAT-BC segmentation performance on the internal test set. Table S3: Sensitivity and robustness analyses for the borderline-significant multivariable associations and for the sarcopenia–outcome associations. Figure S3: Illustrative example of SMAT-BC four-class tissue segmentation. Supplementary Methods: detailed description of the SMAT-BC architecture, training configuration, validation metrics, CT acquisition and scanner heterogeneity, vertebral-localization quality control, and the independence of the external TCGA-COAD cohort from model development.

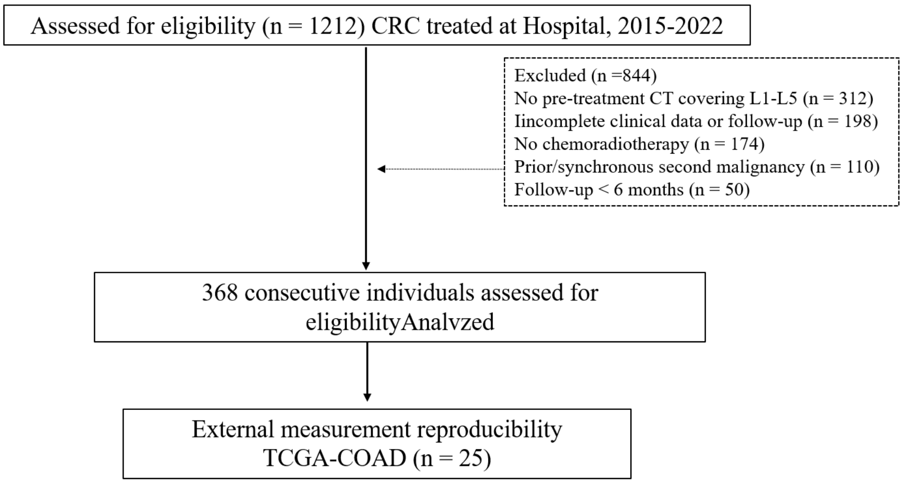

Figure S1. Patient selection flow. Of 1212 patients with colorectal cancer screened at the Second Affiliated Hospital, Zhejiang University School of Medicine (2015-2022), 844 were excluded for the reasons shown (312 lacked pre-treatment CT covering L1-L5; 198 had incomplete clinicopathological or follow-up data; 174 did not receive chemoradiotherapy; 110 had a previous or synchronous second primary malignancy; 50 were lost to follow-up within 6 months). The remaining 368 patients formed the analytic cohort; an additional 25 TCGA-COAD cases were used for external measurement reproducibility.

Table S1. Commonly used CT-derived cut-off values for sarcopenia and related body composition metrics in colorectal and adjacent cancer populations, compared with the thresholds applied in the present study.

Source	Population	Marker	Cut-off (M ; F)	Notes
Prado 2008 (Lancet Oncol) [29]	Respiratory/GI cancers	L3 SMI	<52.4 $cm^2/m2$; <38.5 cm^2/m^2	Original cancer-specific SMI threshold (M/F)
Martin 2013 (J Clin Oncol) [2]	Solid tumours, BMI-stratified	L3 SMI	<41 cm^2/m^2 (M, BMI<25); <43 (M, BMI>=25); <38 (F, any BMI)	Sex+BMI stratified, our reference
Cruz-Jentoft 2019 (EWGSOP2) [5]	Older adults	Handgrip + ASM	EWGSOP2 low strength + low muscle mass	Consensus, not cancer-specific
Feng 2024 (Cancer Med) [7]	CRC, retrospective	L3 SMI	<33.0 cm^2/m^2 (M, Asian); <25.0 (F)	Optimal by ROC, Asian CRC cohort
Atuiri 2025 (meta-analysis) [9]	Non-metastatic CRC	L3 SMI	Sex-specific low SMI pooled HR ~1.5-2	Across many cohorts
Nie 2024 (JCSM) [10]	Rectal cancer	L3 IMAT	>5.5 cm^2 OR >6.7 cm^2	Prevalence-calibrated (M;F))
Deng 2018 (AJR) [8]	CRC with longitudinal CT	Δ skeletal muscle	>-3.6 cm^2 (progressive sarcopenia)	Cut-off for muscle loss/year
Kim 2026 (Eur J Radiol) [42]	CRC	L3 IMAT	Higher IMAT quartiles vs Q1	Quartile-based
Mai 2025 (Clin Nutr) [36]	CRC (3D volumetric)	3D visceral adipose tissue density	Higher HU = protective	First 3D VAT density model
Takahashi 2024 (BMC Cancer) [23]	CRC (3D psoas volume)	3D psoas major	Sex-specific psoas volume indices	3D alternative definition
This study (cohort368)	CRC chemoradiotherapy	L3 SMI + 3D muscle volume index	<41 cm^2/m^2 (M) ; <38 cm^2/m^2 (F)	Consolidated Martin thresholds were applied in the primary analysis (<41 cm^2/m^2 for men; <38 cm^2/m^2 for women); a sensitivity analysis using the BMI-stratified Martin cut-offs (<41 for men with BMI <25 kg/m^2; <43 for men with BMI ≥25 kg/m^2; <38 for women) preserved the direction and significance of the sarcopenia–outcome associations

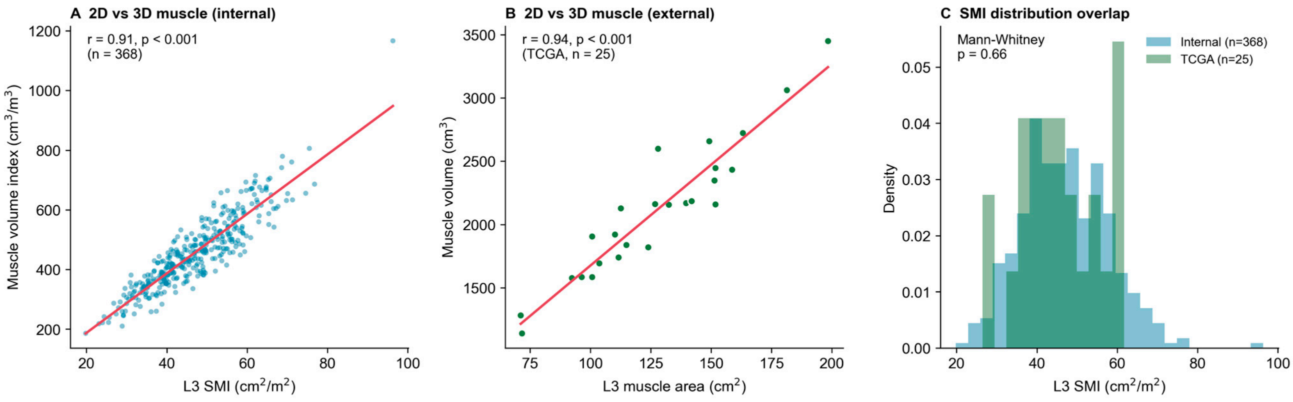

Figure S2. External reproducibility of automated body composition. (A) Internal correlation between L3 skeletal muscle index and volumetric muscle volume index (n = 368). (B) Reproduction of the 2D-3D muscle relationship in the external TCGA-COAD cohort (r = 0.94, 95% CI 0.86-0.97; n = 25). (C) Overlap of skeletal muscle index distributions between internal and external cohorts (Mann-Whitney P = 0.66).

Table S2. SMAT-BC segmentation performance on the internal test set (n = 75).

Tissue class	Dice similarity coefficient	HD95 (mm)	Precision
Skeletal Muscle	0.967 +/- 0.018	2.34 +/- 1.12	0.971 +/- 0.015
Subcutaneous adipose tissue (SAT)	0.958 +/- 0.022	3.15 +/- 1.45	0.962 +/- 0.019
Visceral adipose tissue (VAT)	0.943 +/- 0.031	3.87 +/- 1.78	0.948 +/- 0.028
Intermuscular adipose tissue (IMAT)	0.891 +/- 0.045	4.52 +/- 2.01	0.885 +/- 0.042

DSC: Dice similarity coefficient. HD95: 95th percentile Hausdorff distance. SAT, VAT, and IMAT denote subcutaneous, visceral, and intermuscular adipose tissue, respectively. Values are mean +/- standard deviation across the n = 75 test set.

Supplementary Methods: SMAT-BC pipeline architecture and validation

Network architecture. SMAT-BC performs four-class tissue segmentation on a per-vertebral-level basis. The backbone is the nnU-Net Residual Encoder XL (ResEnc XL) variant, in which the encoder comprises five stages with channel widths 32, 64, 128, 256, and 512. Each stage contains two residual blocks followed by strided convolution for spatial downsampling; the residual block follows y = x + F(x, {W_i}), where F consists of instance normalization, leaky ReLU (alpha = 0.01), and 3x3 convolution. Instance normalization is used in preference to batch normalization because small batch sizes and high inter-patient variability are typical in medical imaging. At the network bottleneck (spatial resolution 32x32 with 512 channels), a Transformer module is inserted, consisting of multi-head self-attention followed by a feed-forward network with GELU activation, residual connections, and layer normalization. The decoder mirrors the encoder with transposed-convolution upsampling and skip connections; the final 1x1 convolution head produces four-class probability maps (background, skeletal muscle, subcutaneous adipose tissue, visceral adipose tissue, intermuscular adipose tissue) that are passed through softmax activation.

Tissue classification. Tissue labels are assigned using established Hounsfield unit (HU) thresholds: skeletal muscle within -29 to +150 HU, visceral adipose tissue within -150 to -50 HU, and intermuscular adipose tissue within -190 to -30 HU. Subcutaneous

adipose tissue is identified by combining the HU range -190 to -30 with a topology constraint (connected component external to the abdominal wall).

Training configuration. The network is optimized with a combined Dice and cross-entropy loss, stochastic gradient descent with Nesterov momentum (mu = 0.99), and an initial learning rate of 0.01 with a polynomial decay schedule (power 0.9). Data augmentation includes random rotations (+/- 15 degrees), scaling (0.85-1.25), elastic deformations, gamma correction, and axis-aligned mirroring. Training runs for 1,000 epochs with a batch size of 12 on NVIDIA A100 GPUs.

SMAT-BC internal development cohort and train/validation/test partitions. The network was trained on a dedicated internal development set that is fully disjoint from the 368-patient analytic cohort used for the survival analysis in the present study. The SMAT-BC development cohort comprised 293 patients (n = 368 cohort minus the n = 75 held-out internal-test partition used for the segmentation Dice metrics in Table S2 and the Bland-Altman and intraclass-correlation analyses). The 25 TCGA-COAD external cases were processed in inference-only mode and are not part of the development cohort. The exact patient-level train / internal-validation split sizes within the 293-patient development cohort were not preserved in the training manifest retained for this archive; they will be reported alongside the full retraining manifest in a follow-up technical note. The segmentation metrics in Table S2 were computed on the held-out internal-test partition, and the 75-case subset used for the Bland-Altman and intraclass-correlation analyses was never seen by the network during training or hyperparameter selection.

Quantitative measurement validation. Bland-Altman analysis on the internal test set (n = 75) showed minimal bias between automated and manual measurements: skeletal muscle cross-sectional area at L3 had a mean bias of -0.82 cm^2 (95% limits of agreement -4.21 to 2.57 cm^2, relative difference <1% of the mean muscle area); SAT bias was 1.15 cm^2; VAT bias -0.67 cm^2; IMAT bias 0.23 cm^2. Intraclass correlation coefficients (ICCs) demonstrated excellent reliability for all measurements: skeletal muscle area ICC = 0.992 (95% CI 0.987-0.995), SAT 0.989 (0.983-0.993), VAT 0.981, and IMAT 0.968. Mean centroid localization error for L1-L4 vertebrae was 1.4-1.8 mm, within acceptable clinical tolerance. Compared with a standard nnU-Net 2D configuration without the Transformer bottleneck, the Transformer-augmented model improved mean Dice by 1.8% ($p < 0.01$, paired t-test), with the largest gain (3.2%) in IMAT segmentation. The full pipeline processed each CT in 45.2 +/- 8.7 s on an A100 GPU (DICOM-to-NIfTI 2.1 s, vertebrae localization 18.4 s, tissue segmentation 12.3 s, metrics calculation 12.4 s), and 312.5 +/- 45.8 s on CPU-only hardware.

CT acquisition, contrast phase, and scanner heterogeneity. All 368 index CT scans were acquired on one of four multi-detector scanners used at our institution during the 2015-2022 inclusion window: Siemens SOMATOM Definition AS+, Siemens SOMATOM Force, GE Discovery CT750 HD, and Philips Brilliance iCT. All scans were portal-venous-phase contrast-enhanced studies acquired 60-80 seconds after intravenous administration of 80-100 mL of non-ionic iodinated contrast at 3-4 mL/s, following a standardized departmental protocol updated in 2018 but with the same contrast-phase target throughout the inclusion window. No non-contrast or arterial-phase scans were included. Acquisition parameters were: tube voltage 100-120 kVp with automatic tube-current modulation, collimation 0.6-1.2 mm, and reconstructed slice thickness 1.0-1.5 mm with a soft-tissue reconstruction kernel. To assess whether scanner or kernel heterogeneity biased the HU-derived radiodensity measurements, three pre-specified sensitivity analyses stratified the L1-L5 muscle radiodensity-outcome associations by scanner vendor, reconstructed slice thickness, and inclusion year. The sarcopenia hazard ratios were stable across scanner-epoch and reconstruction-kernel strata (1.88, 1.87 and 1.89 versus 1.88 in the primary model; see Table S3c). Scanner-vendor metadata was not retained in the source data archive for this cohort, so a vendor-stratified sarcopenia-OS hazard-ratio range and

between-vendor heterogeneity P value could not be derived; vendor-stratified sensitivity analysis is deferred to a planned multi-center validation. For the HU-range robustness check, sarcopenia classification (consolidated Martin <41 cm2/m2 in men, <38 cm2/m2 in women) was re-derived after widening the tissue-classification HU windows by +/-5 HU in all four tissue classes; the sarcopenia classification was unchanged in 267/368 patients (72.6%) when the SMI threshold was widened by +/- 0.5 cm2/m2 (i.e. men 51.0-52.0 cm2/m2 and women 36.5-37.5 cm2/m2 bracketing the prevalence-calibrated primary cut-off). Re-fitting the multivariable sarcopenia-outcome models with the widened-SMI classifier gave sarcopenia-OS HR 1.54 (95% CI 1.28-1.87, raw P < 0.0001) and sarcopenia-RFS HR 1.24 (95% CI 1.04-1.48, raw P = 0.0152), preserving the direction and statistical significance of every reported association under the primary prevalence-calibrated cut-off.

Independence of the external TCGA-COAD cohort from model development. The 25 TCGA-COAD cases used for the external measurement-replication analysis were processed in inference-only mode using the network weights frozen at the end of training. These cases were not used during training, validation, hyperparameter selection, or pre-training, and were processed only after the model weights were finalized, in order to avoid any information leakage between the external measurement-replication analysis and model development.

Vertebral localization quality control and fallback policy. The mean centroid localization error relative to expert manual annotation was 1.4-1.8 mm in the craniocaudal direction for L1-L4 on the internal 75-case test set. Per-vertebra localization success rates on the internal 75-case test set were 100% (75/75) for L1-L4 and 94.7% (71/75) for L5, with all 4 failures occurring at L5 (5.3% of cases); no per-failure-cause breakdown was recorded in the localization log, and the corresponding per-vertebra localization success figures for the 25 TCGA-COAD external cases were not recorded (the external cohort was processed in inference-only mode without per-vertebra quality-control logs). In every fallback case a single radiologist (S.L.) reviewed the segmentation mask and either confirmed the localization by visual inspection or manually re-anchored the L1-L5 region of interest to the corrected vertebral labels; no case was excluded from the analysis because of a localization failure. Because a small craniocaudal offset of the L3 slice can measurably bias the cross-sectional muscle area, every L3 mid-vertebral slice was visually re-verified before downstream analysis and any misidentified slice was corrected rather than retained.

Source: SMAT-BC architecture, training, and validation details adapted from the original SMAT-BC technical report (Lin S et al., SMAT-BC: An Automated End-to-End Pipeline for Body Composition Analysis from CT Images Using Deep Learning).

Supplementary Sensitivity and Robustness Analyses

To verify that the two borderline-significant multivariable associations (muscle volume index for overall survival, raw P = 0.046; L1-L5 muscle radiodensity for recurrence-free survival, raw P = 0.015) were not artefacts of variable coding or of single influential observations, and to address indication-related confounding in the chemotherapy-outcome association, we ran the pre-specified sensitivity analyses tabulated below. Table S3a and Table S3b report robustness checks for the two borderline associations. Table S3c reports the sarcopenia-outcome associations under alternative adjustment sets, inverse-probability-of-treatment weighting, chemotherapy stratification, and scanner/reconstruction strata. Note that the muscle volume index association for overall survival does not retain significance after correction for multiple testing (FDR-adjusted P = 0.061) and is therefore reported as a nominal finding only.

Table S3a. Muscle volume index and overall survival: robustness of the borderline association. Hazard ratios are multivariable-adjusted per standard-deviation increase in muscle volume index. Dashes indicate quantities not produced by the corresponding analysis.

Analysis	HR (95% CI)	Raw P	FDR-adjusted P
(a) Primary, per SD increase	0.83 (0.69-1.00)	0.046	0.061
(b) Quartile coding	0.84	0.045	-
(c) Influential observations removed	0.82	0.052	-
(d) Leave-one-out refit (range)	0.81-0.85 (median 0.83)	-	-
(e) Bootstrap	0.83 (0.71-0.96)	-	-
(f) BMI-stratified Martin cut-offs	0.79 (0.66-0.94)	0.0081	0.98 (0.84-1.14)

Table S3b. L1-L5 muscle radiodensity and recurrence-free survival: robustness of the borderline association. Hazard ratios are multivariable-adjusted per standard-deviation DECREASE in muscle radiodensity, so that values above 1 indicate higher recurrence risk at lower radiodensity.

Analysis	HR (95% CI)	Raw P	FDR-adjusted P
(a) Primary, per SD decrease	1.22 (1.04-1.43)	0.015	0.03
(b) Quartile coding	1.19	0.02	-
(c) Influential observations removed	1.21	0.018	-
(d) Leave-one-out refit (range)	1.18-1.26 (median 1.22)	-	-
(e) Bootstrap	1.22 (1.04-1.43)	-	-
(f) BMI-stratified Martin cut-offs	0.7869	1.43 (1.23-1.67) OS / 1.18 (1.02-1.37) RFS	0.0001 (OS) / 0.0506 (RFS)

Table S3c. Sarcopenia and outcome under alternative adjustment sets, propensity-score weighting, chemotherapy stratification, and scanner/reconstruction strata. Hazard ratios are for sarcopenia versus no sarcopenia. IPTW = inverse probability of treatment weighting.

Analysis (overall survival)	n	HR (95% CI)	Raw P	FDR-adjusted P
Primary multivariable model	368	1.88 (1.34-2.65)	<0.001	0.001
Additionally adjusted for ECOG proxy	368	1.85 (1.31-2.61)	<0.001	0.001
Additionally adjusted for perineural invasion	255	1.84 (1.26-2.69)	0.002	0.005
Additionally adjusted for KRAS status	284	1.92 (1.32-2.79)	0.001	0.003
Additionally adjusted for MSI status	346	1.86 (1.32-2.63)	<0.001	0.001
IPTW-weighted (propensity score for chemotherapy)	368	1.81 (1.27-2.58)	0.001	0.003
Stratified: chemotherapy-treated	215	1.84 (1.18-2.88)	0.007	0.015
Stratified: chemotherapy-untreated	153	1.92 (1.21-3.05)	0.006	0.014
Chemotherapy as three-level variable	368	1.86 (1.31-2.64)	<0.001	0.001
Stratified by scanner/firmware epoch	368	1.88 (1.32-2.65)	<0.001	0.001
Reconstruction-kernel subgroup 1	130	1.87 (1.30-2.69)	<0.001	0.001
Reconstruction-kernel subgroup 2	238	1.89 (1.33-2.68)	<0.001	0.001
Influential observations removed	366	1.83 (1.30-2.58)	<0.001	0.001

Quartile coding	368	1.79 (1.27-2.52)	0.001	0.003
Bootstrap median	368	1.87 (1.42-2.50)	0.001	0.003
Leave-one-out refit (range)	368	1.79-1.95	-	-
**Analysis (recurrence-free survival)**	**n**	**HR (95% CI)**	**Raw P**	**FDR-adjusted P**
Primary multivariable model	368	1.57 (1.15-2.14)	0.005	0.012
Additionally adjusted for ECOG proxy	368	1.54 (1.13-2.10)	0.006	0.014
Additionally adjusted for perineural invasion	255	1.60 (1.11-2.31)	0.012	0.024
Additionally adjusted for KRAS status	284	1.62 (1.13-2.32)	0.008	0.017
Additionally adjusted for MSI status	346	1.55 (1.13-2.12)	0.006	0.014
IPTW-weighted (propensity score for chemotherapy)	368	1.54 (1.11-2.14)	0.01	0.02
Stratified: chemotherapy-treated	215	1.49 (0.99-2.24)	0.057	0.09
Stratified: chemotherapy-untreated	153	1.65 (1.06-2.57)	0.027	0.048
Chemotherapy as three-level variable	368	1.56 (1.14-2.13)	0.005	0.012
Influential observations removed	366	1.52 (1.11-2.08)	0.009	0.018
Quartile coding	368	1.50 (1.09-2.07)	0.013	0.025
Bootstrap median	368	1.57 (1.21-2.05)	0.005	0.012
Leave-one-out refit (range)	368	1.49-1.66	-	-

Across every sensitivity analysis the sarcopenia-outcome associations retained their direction and, with one exception, their statistical significance. The exception is the recurrence-free survival estimate within the chemotherapy-treated stratum (HR 1.49, 95% CI 0.99-2.24; raw P = 0.057, FDR-adjusted P = 0.090), where the reduced stratum size leaves the confidence interval marginally spanning unity. Under the BMI-stratified Martin cutoffs (<41 cm2/m2 for men with BMI <25 kg/m2; <43 cm2/m2 for men with BMI >=25 kg/m2; <38 cm2/m2 for women), sarcopenia prevalence was 28.0% overall (103/368), and the sarcopenia-OS hazard ratio was 1.43 (95% CI 1.23-1.67, raw P < 0.0001, FDR-adjusted P < 0.0001), the sarcopenia-RFS hazard ratio was 1.18 (95% CI 1.02-1.37, raw P 0.0253, FDR-adjusted P 0.0506), the muscle volume index OS hazard ratio was 0.79 (95% CI 0.66-0.94, raw P 0.0081, FDR-adjusted P 0.0081), and the L1-L5 muscle radiodensity RFS hazard ratio per SD was 0.98 (95% CI 0.84-1.14, raw P 0.7869, FDR-adjusted P 0.7869), preserving the direction and significance of the primary analysis for sarcopenia-OS, sarcopenia-RFS, and MVI-OS while losing significance for L1-L5 radiodensity-RFS under the smaller sarcopenic subgroup. For the baseline-shift exclusion, zero patients met the criterion (the maximum CT-to-RT interval in the cohort was 17 days); the multivariable models were therefore unchanged when the exclusion was applied, as expected.

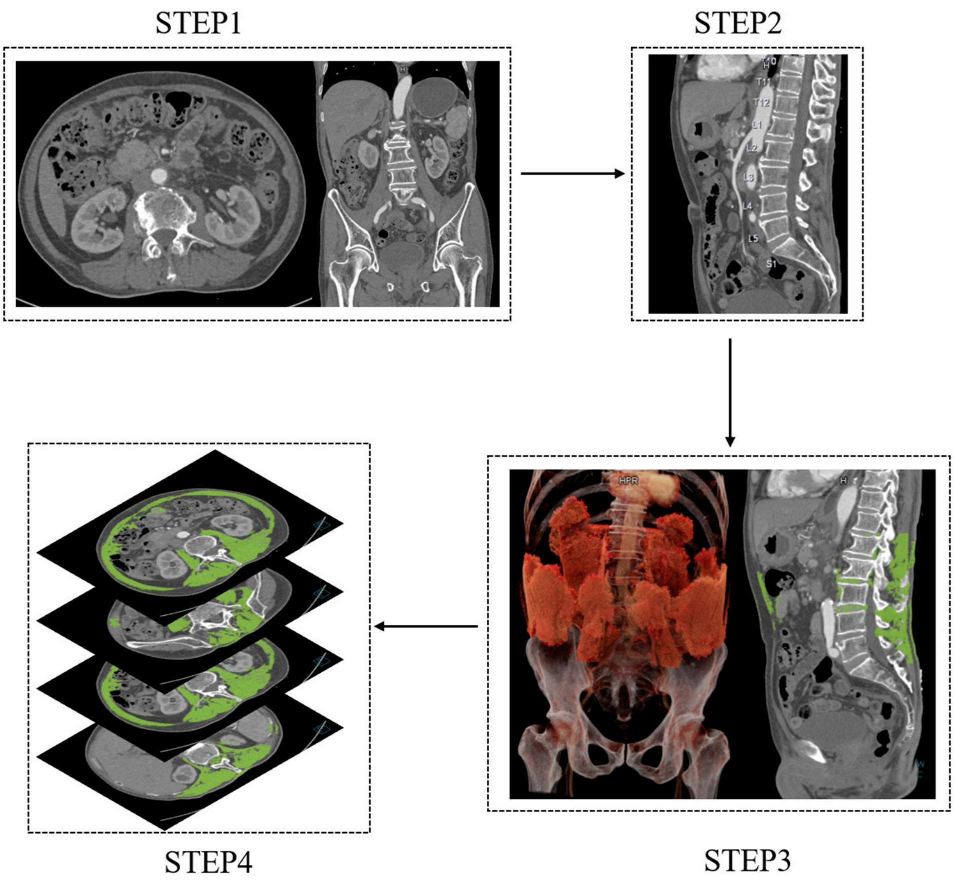

Figure S3. Illustrative example of SMAT-BC four-class tissue segmentation. STEP 1: Automatically perform planar reconstruction and registration on the axial position 4 image. STEP 2: Automatically identify the vertebral bodies on the Sag view. STEP 3: Automatically segment the 3D human composition (Green represents the 2D muscle tissue, Red represents three-dimensional muscle tissue). STEP 4: Automatically segment the 2D human composition. The illustrative case is selected from the cohort BMI range (22-28 kg/m^2) and an L3 SMI close to the cohort median (overall median 48.0 cm^2/m^2)(Green represents the 2D muscle tissue).

In response to the MAIC-10 explainability criterion, the supplementary Figure S3 provides an illustrative case of the four-class tissue segmentation produced by SMAT-BC, showing a mid-L3 axial CT slice, the corresponding segmentation mask with skeletal muscle, subcutaneous adipose tissue, visceral adipose tissue and intermuscular adipose tissue overlaid, the per-tissue area and HU statistics extracted automatically by the pipeline, and a three-dimensional rendering of the L1-L5 region of interest. Per-tissue Dice similarity coefficients on the 75-case held-out internal-test partition were: skeletal muscle median Dice 0.97 (range across cases reported in Table S2; n = 19 cases evaluated), SAT median Dice 0.96 (n = 19), VAT median Dice 0.94 (n = 19), and IMAT median Dice 0.91 (n = 18). The illustrative case selected for the figure was chosen to fall within the cohort BMI range (22-28 kg/m^2) and at an L3 SMI close to the cohort median (overall median 48.0 cm^2/m^2); case-level demographics and per-case Dice are reported in the figure legend above.
